# The Origin of the Feedstock Molecules for Life on the Hadean Earth

**DOI:** 10.1002/anie.202512374

**Published:** 2025-09-01

**Authors:** Nikolai Diukarev, Erik Boinowitz, Jonas Feldmann, Stefan Wiedemann, Aleksandr Mikheev, Sidney Becker, Thomas Carell

**Affiliations:** ^1^ Department of Chemistry Institute for Chemical Epigenetics, Ludwig‐Maximilians‐Universität München Würmtalstraße 201 81375 Munich Germany

**Keywords:** Amino acids, Nucleosides, Origin of life, Oxime, Prebiotic chemistry

## Abstract

The origin of life in the Hadean eon required the formation of amino acids and nucleosides among the key molecules of life. It is assumed that highly unsaturated carbon and nitrogen containing feedstock molecules, which form in a strongly reducing H_2_‐atmosphere were the reactive starting materials. The Hadean Earth was, however, surrounded by a CO_2_‐dominated atmosphere and the question of how under such inert conditions the biomolecules could have formed is unsolved. Here, we report that HNO and formaldehyde, which form robustly in a weakly reducing atmosphere by lightning and UV‐irradiation, react with SO_2_ to produce oxime, amidoxime, and nitrile‐containing molecules, which react to give precursors to amino acids and all four canonical nucleosides. The small number of feedstock molecules required suggests that the molecules of life can robustly form on all types of rock planets with volcanic activity and a CO_2_‐dominated atmosphere.

## Graphical abstract

The origin of life in the Hadean eon required the formation of amino acids and nucleosides among the key molecules of life. In an often postulated strongly reducing atmosphere, HCN and its derivatives are robustly formed as plausible starting molecules. But HCN does not form in a Hadean, weakly reducing atmosphere. Instead, aldehydes and NO are produced, which react to produce oxime, amidoxime, and nitrile‐based feedstock molecules as precursors.

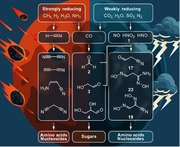

## Introduction

Life started on the Hadean Earth around 4 billion years ago and this required reactive feedstock molecules, from which lipids, amino acids, sugars, and nucleosides eventually formed, either directly^[^ ^1^, ^2^ ^]^ or via prebiotic metabolic circuits.^[^ ^3^, ^4^ ^]^ The amino acids and nucleosides must then have reacted to peptides and oligonucleotides as the essential biopolymers, which finally allowed life to emerge.^[^ ^1^, ^2^, ^5^, ^6^ ^]^ The late Hadean Earth had a weakly reducing atmosphere composed mainly of CO_2_, H_2_O, and N_2_, with potentially small concentrations of H_2_, NH_3_, CH_4_, and CO.^[^ ^7^, ^8^, ^9^, ^10^ ^]^ The dominant sulfur gas was probably SO_2_, emitted into the atmosphere by volcanic activity.^[^ ^11^, ^12^, ^13^ ^]^ Lightning and UV‐irradiation, together with heat from volcanic activity and the presence of reducing power from either dissolved ferrous ions (Fe^2+^)^[^ ^14^, ^15^ ^]^ or atmospheric SO_2_ must have provided the energy to fuel these early prebiotic reactions. Although Miller–Urey type experiments^[^ ^16^, ^17^ ^]^ under CO_2_‐dominated atmospheric conditions indeed provided amino acids, neither the formed intermediates nor the chemical pathways are known.

**Figure 1:**
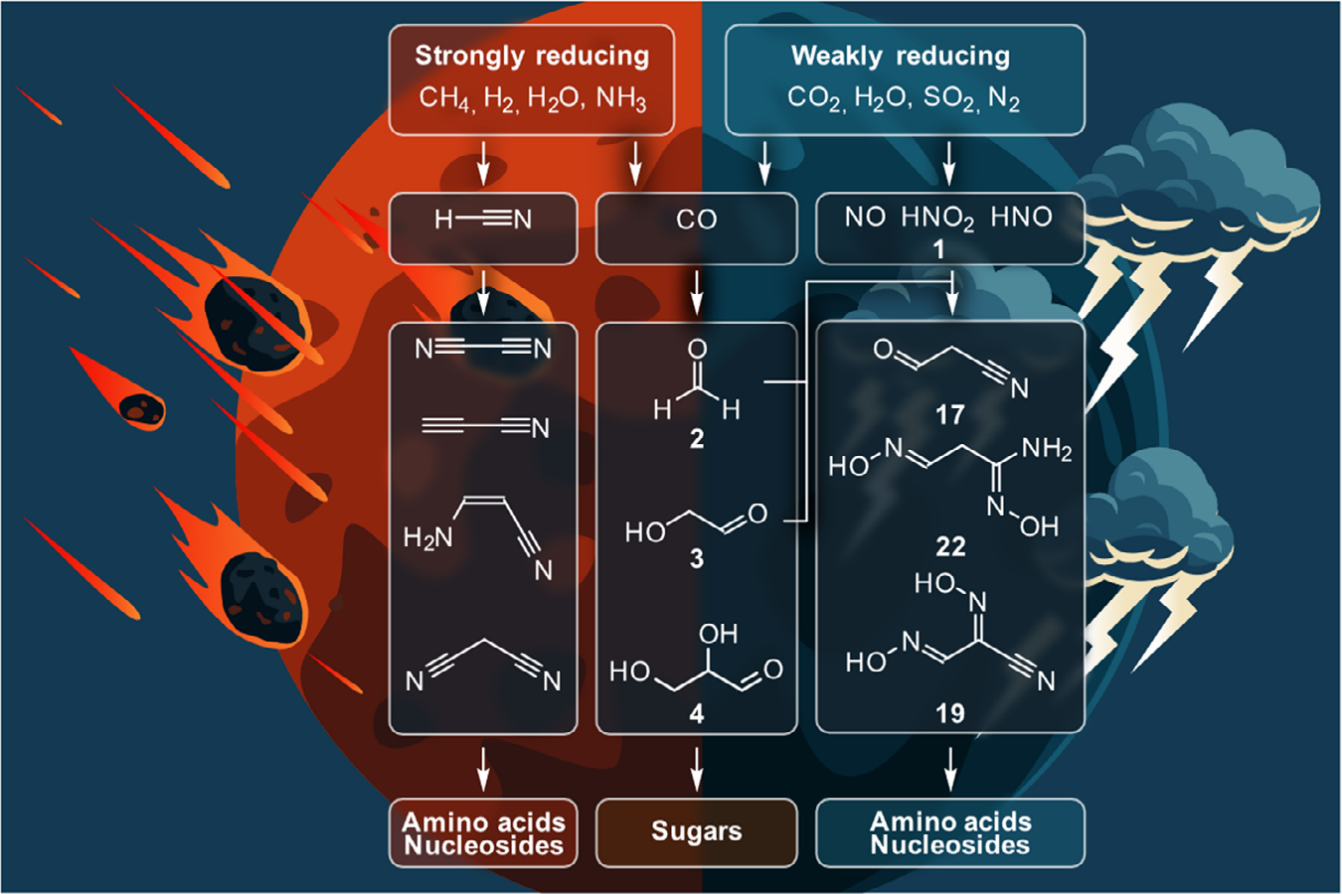
Formation of feedstock molecules under strongly reducing (H_2_‐dominant) and weakly reducing (CO_2_‐dominant) atmospheric conditions. While in a strongly reducing atmosphere HCN and its derivatives are robustly formed as plausible starting molecules towards amino acids and nucleosides, HCN does not form in a weakly reducing, CO_2_‐dominated atmosphere. Under such conditions, aldehydes and NO are produced, which react to produce oxime, amidoxime, and nitrile‐based feedstock molecules, which then allow the formation of amino acids and nucleosides.

The currently dominating chemical ideas about how the biomolecules could have formed on the Hadean Earth are based on HCN and feedstock molecules derived from HCN such as cyanogen, cyanoacetylene, 3‐aminoacrylonitrile, and malononitrile (Figure 1).^[^ ^1^, ^2^, ^5^, ^18^ ^]^ This requires the robust formation of atmospheric HCN, which is only possible in a strongly reducing H_2_‐dominated atmosphere.^[^ ^1^, ^19^, ^20^, ^21^, ^22^, ^23^ ^]^ Concepts of how to create HCN in a CO_2_‐dominated Hadean atmosphere included a complete or partial shift of the atmosphere into a H_2_‐state by an impact event, which would not have lasted until the late Hadean.^[^ ^24^, ^25^, ^26^ ^]^ The local formation of HCN by aerodynamic ablation of carbonaceous chondrites has also been suggested.^[^ ^27^ ^]^ The potential liberation of large amounts of HCN from ferrocyanide deposits is also discussed.^[^ ^28^, ^29^, ^30^ ^]^ Finally, an exogenous delivery of the feedstock molecules to the early Earth by meteorites was suggested.^[^ ^31^ ^]^

While all these concepts rescue this HCN based prebiotic chemistry, a more stringent origin of life scenario would ask for concepts that allow the formation of feedstock molecules without atmospheric HCN. The problem with origin of life concepts based on atmospheric HCN is mainly that the needed H_2_‐atmosphere is only stable for a short period of time due to rapid H_2_ loss from Earth, which limits the time span available for the formation of the biomolecules. In addition, CO_2_‐atmospheres were recently detected on exoplanets. Hence alternative CO_2_‐based chemical scenarios are also needed to predict the potential for life in other solar systems.^[^ ^32^ ^]^ Here, we report chemical pathways that are compatible with an atmosphere composed of CO_2_, N_2_, H_2_O, and SO_2_. This chemistry generates a small set of oxime‐ and nitrile‐containing feedstock molecules (Figure 1) as parent molecules for amino acids and all four nucleosides along coupled prebiotic reaction sequences.

## Results and Discussion

### Formation of Sugars and Amino Acids in a Weakly Reducing Atmosphere

We first revisited which fundamental molecules form under such conditions (Figure 1). Lightning through such an atmosphere is known to generate nitrogen monoxide NO (Supporting Information, S1.1).^[^ ^16^, ^19^, ^33^, ^34^, ^35^, ^36^, ^37^ ^]^ Calculations predict that about 0.5–0.7 million tons of NO were potentially generated per year on the Hadean Earth.^[^ ^33^, ^34^ ^]^ NO reaches the surface as HNO, which in water produces NO_2_^−^ (**1**) and NO_3_^−^ (Supporting Information, S1.2).^[^ ^35^, ^36^, ^37^ ^]^ It was estimated that the NO_x_^−^ concentration in the Hadean oceans could have reached concentrations of >20 mM.^[^ ^36^ ^]^ Even higher concentrations were suggested for shallow ponds.^[^ ^37^ ^]^ Important is the effect that UV irradiation converts NO_3_^−^ to NO_2_^−^ (**1**).^[^ ^36^, ^37^ ^]^

**Figure 2:**
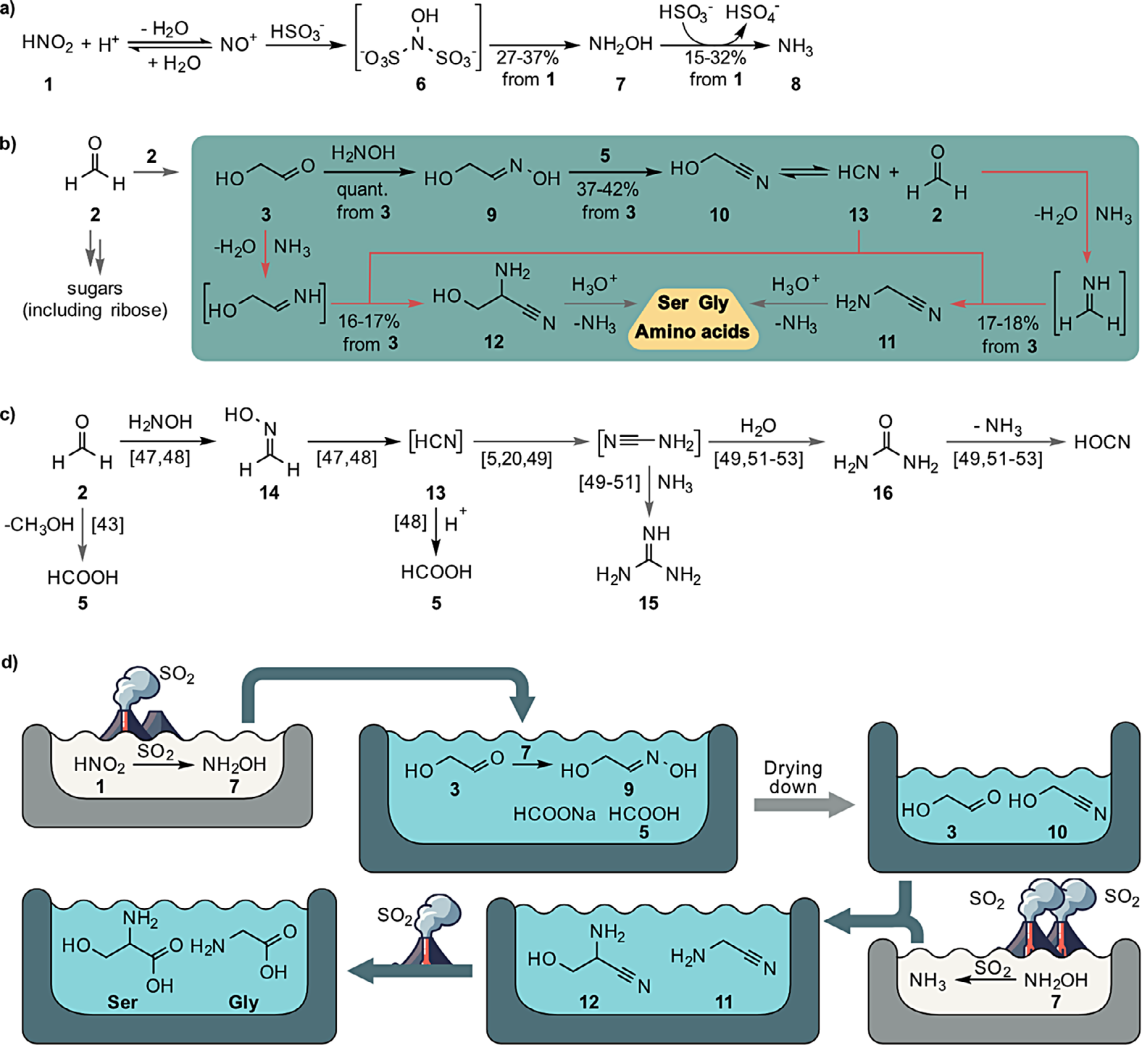
Prebiotic formation of feedstock molecules and their reactions towards amino acids. Gray arrows represent reactions taken from literature and black arrows represent reactions performed in this work. a) Reaction of HNO_2_ (**1**) formed from NO with volcanic SO_2_ for N‐fixation leads to hydroxylamine (**7**) and ammonia (**8**). b) A chemical scenario, in which prebiotically plausible glycolaldehyde reacts with hydroxylamine (**7**) and ammonia (**8**) to produce oxime feedstock molecules from which α‐aminonitriles such as **11** and **12** and then amino acids such as Gly and Ser can form. The red lines show the putative Strecker pathway involving reactions of **10** to **11** and **3** to **12** under intermediate formation of HCN (**13**). c) HCN (**13**) can also form in a dry‐down reaction via the elimination of water from formaldoxime (**14**). This process can provide further feedstock molecules such as formic acid (**5**), guanidine (**15**) urea (**16**) and other amidinium compounds along published pathways.^[^ ^51^, ^52^, ^53^, ^54^, ^55^ ^]^ d) Connected shallow pond model for the simultaneous formation of Gly and Ser from glycolaldehyde (**3**).

It is furthermore known that UV irradiation of such a CO_2_‐dominated atmosphere generates carbon monoxide (CO) and formaldehyde (**2**) (Supporting Information, S1.3).^[^ ^38^, ^39^ ^]^ It was estimated that per year about 3 million tons of **2** were potentially removed from the Hadean atmosphere into the Hadean oceans.^[^ ^39^ ^]^ Formaldehyde (**2**) is a plausible starting material for the formation of glycolaldehyde (**3**)^[^ ^40^ ^]^ and glyceraldehyde (**4**),^[^ ^41^, ^42^ ^]^ which were likely the key molecules for the formation of sugars such as ribose.^[^ ^40^, ^41^, ^42^, ^43^ ^]^ Towards the selective formation of ribose, it was found that borate stabilizes pentoses, which could have established a ribose enrichment process.^[^ ^43^, ^44^ ^]^ For the formose reaction, which is often discussed as the basis reaction to give ribose, it was recently demonstrated that the reaction yields mainly branched ketoses.^[^ ^45^ ^]^ Hence, the origin of ribose on the early Earth remains an unsolved problem. Important for this study is that formaldehyde (**2**) disproportionates to formic acid (**5**) and methanol (Figure 2c).^[^ ^46^ ^]^

SO_2_ was likely present in the Hadean atmosphere from volcanic activity. It gives bisulfite (HSO_3_^−^) in water. Models suggest millimolar concentrations of bisulfite on the Hadean Earth,^[^ ^12^ ^]^ which could have stabilized aldehydes (**3** and **4**) as their bisulfite adducts (**3**@**B**, **4**@**B**, Supporting Information).^[^ ^44^ ^]^

**Figure 3:**
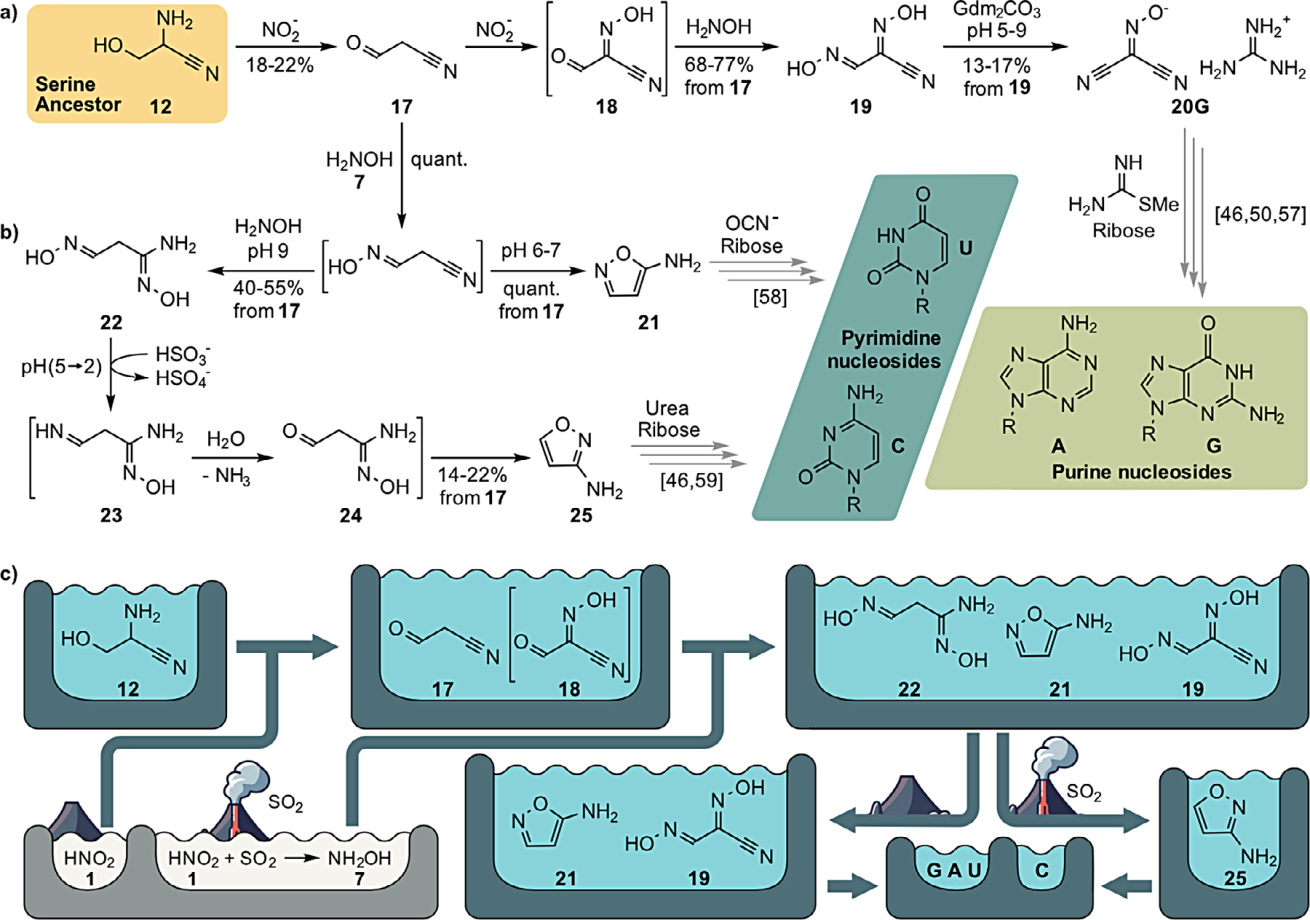
Prebiotic formation of purine and pyrimidine nucleosides. R = ribosides (mixture of α,β‐pyranosides and α,β‐furanosides). Gray arrows represent reactions taken from the literature and black arrows represent reactions performed in this work. a) Formation of purine nucleosides from cyanoacetaldehyde (**17**) with first no volcanic activity and hence the presence of only NO_2_^−^, followed by volcanic activity that allows the formation of NH_2_OH (**7**). b) Formation of pyrimidine nucleosides from **17** with immediately switched‐on volcanic activity that generates SO_2_ and therefore a high amount of NH_2_OH. Depending on pH, this gives either **21** (pH 6–7) or **25** (pH 9). c) A connected pond scenario that provides pyrimidine and purine nucleosides.

In order to study how the fundamental molecules HNO_2_, SO_2_, formaldehyde, and glycolaldehyde (**1**–**3**) could have created feedstock molecules for the formation of amino acids (Figure 2) and nucleosides (Figure 3). In a first experiment (Figure 2a) we allowed a solution of NaNO_2_ (**1·Na**, 20 mM)^[^ ^36^, ^37^ ^]^ to concentrate in a gentle dry‐down step (7 days, 35 °C). We subsequently passed SO_2_ through the solution which allowed the pH to slowly decrease due to the formation of H_2_SO_3_ (pH ≈ 3). Under these conditions, hydroxylamine bisulfonate (**6**) forms initially,^[^ ^47^ ^]^ which hydrolyzes in the acidic environment to hydroxylamine (**7**) (S1.4).^[^ ^48^ ^]^ With the idea in mind that the reaction likely took place close to volcanic activity, where SO_2_ would be readily available, we performed the reaction of NaNO_2_ with SO_2_ at pH ≈ 3 at about 70 °C and obtained hydroxylamine (**7**) with a yield between 27%–37% and ammonia (**8**) with a yield between 15%–32% in just 3 h (S2).

We next repeated the above experiment and simulated higher volcanic activity by passing more SO_2_ through the solution. Now, the pH dropped to pH ≈ 2 and we detected the reduction of hydroxylamine (**7**) to ammonia (**8**) under concomitant formation of SO_3_/H_2_SO_4_ (Figures 2a, S1.5, and S3).

Together, these data show that the amount of SO_2_ controls the ratio between hydroxylamine (**7**) and ammonia (**8**). Importantly, formation of ammonia (**8**) is not only possible from **7** and SO_2_, but via the reduction of NO_x_^−^ with Fe^2^⁺ (Supporting Information, S1.5).^[^ ^15^ ^]^

Towards the formation of amino acids in a CO_2_‐dominated atmosphere, we investigated a scenario (Figure 2b), in which the formaldehyde (**2**) concentration is very low because it disproportionated to methanol and formic acid (**5**).^[^ ^46^ ^]^ Consequently, little glycolaldehyde (**3**) would be present^[^ ^41^, ^42^ ^]^ and as such available hydroxylamine (**7**) will convert all **3** into 2‐hydroxyacetaldehyde oxime (**9**). We therefore treated glycolaldehyde (**3**) with a small excess of NH_2_OH (**7**, 1.2 eq., 25 °C) in the presence of formic acid buffer (**5**, pH ≈ 4) and detected quantitative formation of 2‐hydroxyacetaldoxime (**9**) (Supporting Information, S4). If we imagine that this happened close to volcanic activity generating plenty of SO_2_, **3** will exist as its bisulfite adduct **3**@**B** (Supporting Information). We consequently reacted also the adduct **3**@**B** with NH_2_OH (**7**) in the presence of formic acid (**5**, pH ≈ 4) and found that compound **9** was now generated in yields between 52%–56% (Supporting Information, S5).

In order to study if the present NH_2_OH is compatible with the simultaneous presence of sugars like ribose, we added ribose (1.0 eq.) to the reaction mixture containing **3@B** (1.0 eq.) and NH_2_OH (**7**, 0.6–1.0 eq.). In this case no difference regarding the formation of **9** was observed, likely because NH_2_OH (**7**) reacts first with **3** and **3**@**B**, but not with sugars because the aldehyde group is present as a half‐acetal in the cyclic form (Supporting Information, S6).

Next, we envisioned a formic acid concentration step. This was simulated by gently heating the solution containing glycolaldehyde (**3**) and hydroxylamine (**7**) to moderate 50 °C (1 M formic acid containing buffer). Under these conditions we observed the formation of **9**, but now, due to a concentration of the formic acid (**5**), which forms an azeotrope with water at max. 77.5% **5**, we observed the elimination of water, which converted **9** into the glycolonitrile (**10**), likely after intermediate formation of the formyl ester. We obtained **10** after 2 weeks of slow concentration in yields between 21%–26% (from **3**, Supporting Information, S7). When we simulated a progressed dry‐down and used a more concentrated 5 M formic acid (**5**) containing buffer for the dry‐down step, glycolonitrile (**10**) was obtained from **3** after already 3 days at 50 °C in yields between 34%–38% (Supporting Information, S8). When we performed the reaction, using the oxime **9** as the starting material, **10** was robustly obtained in 37%–42% yield (Supporting Information, S9).

In a scenario, in which a solution of ammonia (**8**), potentially generated in a second pond from SO_2_ and NH_2_OH (**7**), is reaching a pond containing **10** (after connection of these previously separate ponds), the compounds ammonia (**8**) and **10** will first coexist. If we, however, allow the pH to increase due to rising NH_3_ (**8**) concentrations, **10** will be converted to the aminoacetonitrile (**11**). We simulated this idea by adjusting the pH of the solution containing **10** to ≈ 9 upon addition of NH_3_ (**8**) (11 eq. of **8**). Under these conditions the formation of the aminonitrile (**11**) in yields between 34%–35% from **3** (Supporting Information, S10) over 1 day (4 days in total from glycolaldehyde **3**) was observed. If one now imagines a potential outbreak of volcanic activity, so that the SO_2_ concentration increases, providing acidic conditions, hydrolysis **11** to the amino acid glycine (Figure 2b,d) is plausible.

In a second prebiotic scenario towards amino acids (Figure 2b,d), we imagined a shallow pond containing a limiting amount of NH_2_OH (**7**), insufficient to convert all **3** into **9**. Under these conditions (with **5** present), an interesting reaction network is observed. When we treated glycolaldehyde **3** with 0.6 eq. of NH_2_OH (**7**), **9** forms, contaminated with unreacted **3**. After a following gentle dry‐down step with a concentration of formic acid (**5**) (we used 5 M formic acid (**5**) buffer for 3 days at 50 °C), we detected the formation of a solution containing glycolonitrile (**10**) together with unreacted **3**. When we next simulated an influx of NH_3_ (**8**), potentially formed in a second pond, we discovered after 1 day (Supporting Information, S11) the simultaneous appearance of the aminonitriles **11** (17%–18%) and **12** (16%–17%), adding up to a total aminonitrile yield of 33%–35% from glycolaldehyde **3**. We explain the surprising formation of **12** with a decomposition of **10** to formaldehyde (**2**) and HCN (**13**). Formaldehyde (**2**) and leftover glycolaldehyde (**3**) can then react with NH_3_ (**8**) to give the corresponding imines, which react with the liberated HCN (**13**) at pH ≈ 9 in a Strecker reaction to the respective aminonitriles **11** and **12** (Figure 2b,d). We never observed an accumulation of HCN which could interfere with the chemistry.

If we again imagine that at some point the formation of SO_2_ resumes through volcanic activity (Figure 2d), leading to an acidification of the reaction mixture, the aminonitriles **11** and **12** will hydrolyze to the respective amino acids Gly and Ser and ammonia (**8**).

Alternatively, formaldehyde (**2**) will also react with hydroxylamine (**7**), which provides formaldoxime (**14**),^[^ ^49^, ^50^ ^]^ which can eliminate water in a dry‐down reaction to give HCN (**13**).^[^ ^49^, ^50^ ^]^ This HCN (**13**) will then hydrolyze to formic acid (**5**) and ammonia (**8**) (Figure 2c).^[^ ^50^ ^]^ Importantly, the formed HCN is of course available to start the HCN‐based chemistry that has so far dominated our view of prebiotic chemistry.^[^ ^1^, ^2^ ^]^ Some of the HCN‐based reaction products, such as guanidine (**15**) and urea (**16**) are important downstream molecules needed for the formation of pyrimidines and purines (vide infra). HCN (**13**) is for example an established precursor to cyanamide,^[^ ^20^, ^22^, ^51^ ^]^ which can be converted with leftover NH_3_ (**8**) to guanidine (**15**) (Supporting Information, S1.6).^[^ ^51^, ^52^, ^53^ ^]^ Cyanamide is also known to react with water to urea (**16**) and isocyanate (Supporting Information, S1.6).^[^ ^51^, ^53^, ^54^, ^55^ ^]^ For other amidinium compounds, cyanamide may react with different amines^[^ ^56^ ^]^ or thiols. Alternatively, guanidine (**15**) is known to form from urea (**16**) and ammonia (**8**) on hot SiO_2_ surfaces, which are a hallmark of volcanic activity.^[^ ^57^ ^]^ Guanidine (**15**), urea (**16**), and other potentially formed amidinium compounds are typically stable molecules that can accumulate over time to prebiotically relevant concentrations.^[^ ^58^ ^]^

### Formation of Pyrimidine and Purine Nucleosides

We next explored if serine aminonitrile (**12**) would enable the formation of pyrimidine and purine nucleosides as well, which would establish **12** as a common molecular ancestor (Figure 3).

We first investigated the reaction of **12** in the absence of SO_2_ and hence hydroxylamine (**7**), with just the presence of HNO_2_ (NO^+^). When we (Figure 3a) exposed **12** (20 mM, pH 2–4, 25 °C) to 2 eq. HNO_2_ (**1**), we detected the formation of cyanoacetaldehyde (**17**). When we allowed a more vigorous formation of SO_2_, **17** formed the bisulfite adduct **17**@**B** (not shown in Figure 3 for clarity, included in Supporting Information). We found that **17**@**B** forms under such circumstances in yields between 15%–22% (Supporting Information, S12).

We next studied the reaction of **17**@**B** with 3 eq. of **1** and noted that it reacts with a second molecule of NO_2_^+^ (**1·Na**, pH ≈ 2–5), to give *N*‐hydroxy‐2‐oxoacetimidoyl cyanide **18** (Figure S10b). We did not isolate **18**, but reacted it with NH_2_OH (**7**), which may flow into the pond from a neighboring reservoir after a restart of volcanic activity. This furnishes *N*‐hydroxy‐2‐(hydroxyimino)acetimidoyl cyanide **19** in yields between 68%–77% (Supporting Information, S13). **19** is a molecule with a low p*K*_a_‐value that forms salts, for example with guanidine **19·G** (Figure 2c). We discovered that if **19·G** (20 mM) is gently heated to 50 °C (pH ≈ 5–9, 2 days), one of the oxime groups eliminates water, which generates hydroxycarbonimidoyl dicyanide **20**, which we isolated as its guanidinium salt **20·G** in yields between 13%–17% (Supporting Information, S14). The ability to form **20·G** from **12** with just NO_2_^−^, NH_2_OH (**7**) and guanidinium (**15**) is a fascinating discovery, because **20·G** is an already described starting material for the formation of diaminopurine ribosides and subsequently guanosine.^[^ ^48^, ^52^, ^59^ ^]^ If **20** is alternatively complexed to methyl carbamimidothioate, adenosine is formed.^[^ ^59^ ^]^

Together, these data establish the serine aminonitrile **12** as a parent compound for the amino acid serine and adenosine as well as guanosine.

Towards pyrimidines, we just changed the environmental conditions slightly. We just assumed ongoing volcanic activity making NH_2_OH (**7**) available (Figure 3b). When we reacted cyanoacetaldehyde (**17**@**B**) with 8 eq. NH_2_OH (**7**), we observed the fast and quantitative conversion of **17**@**B** (pH ≈ 6–7, 25 °C, 1d) to 5‐aminoisoxazole **21** (Supporting Information, S15). The ability to generate **21** from **11** with just NO^+^ and NH_2_OH (**7**) is a key discovery, because **21** is a known direct precursor for the pyrimidine nucleoside uridine along prebiotically plausible pathways.^[^ ^60^ ^]^

When we furthermore changed the reaction conditions slightly allowing **17**@**B** to react (pH ≈ 4) first with NaNO_2_ (**1·Na**) and subsequently with NH_2_OH (**7**) (1.25 eq. and 1 h each, 25 °C), we observed the parallel formation of 5‐aminoisoxazole **21** (17%–18%) and *N*‐hydroxy‐2‐(hydroxyimino)acetimidoyl cyanide **19** (24%–27%) (Supporting Information, S16). This observation shows that the direct precursors for the nucleosides A, G, and U can form together in one pond.

Importantly, when we again assumed slightly different conditions and exposed **17**@**B** to NH_2_OH (**7**) at a slightly higher pH (pH ≈ 8–9), possibly through the presence of some ammonia, we detected the parallel formation of *N'*‐hydroxy‐3‐(hydroxyimino)propanimidamide (**22**) (40%–55%) and the uridine precursor 5‐aminoisoxazole (**21**) (40%–60%, Figure S14b). When we now envisioned a further increase of volcanic activity so that **22**/**21** are exposed to an excess of aqueous SO_2_ (**22** plus 4 eq. of NaHSO_3_, pH ≈ 5), we detected reduction of **22** to the imine–oxime **23**, which hydrolyzed to the corresponding aldehyde **24**. Both **23** and **24** form then the corresponding bisulfite adducts **23**@**B** (21%–35%) and **24**@**B** (10%–13%; Supporting Information, S17). Under these acidic conditions, **21** completely hydrolyses first to 2‐cyanoacetamide (40%–60%, Figure S14c) and next to 2‐cyanoacetic acid (Figure S15b–c). A pond containing a mixture of **22**/**21** will therefore finally contain **23**@**B** and **24**@**B** plus 2‐cyanoacetic acid.

Warming of this mixture (50 mM, 3:1 ratio) to 70 °C for 7 days leads to an interesting cyclization, which provides 3‐aminoisoxazole **25** in yields between 35%–40% (Supporting Information, S18). This is another important discovery because **25** is a recently reported precursor for the nucleoside cytidine (C).^[^ ^48^, ^61^ ^]^ We found that compound **25** forms in yields between 14%–22% from **17**@**B** and hence ultimately again from **12**. A by‐product of this reaction is SO_3_ and hence in water H_2_SO_4_, which certainly contributes to the pH decrease to finally pH ≈ 1–2 observed during the reaction.

Altogether, the data show that serine aminonitrile **12** is not only the ancestor molecule for the amino acid serine, but also for the four canonical nucleosides. Important is the discovery that U is not (only) derived from C by hydrolysis, but that both pyrimidine nucleosides are generated independently from the same feedstock molecule **17**, along reaction pathways that differ just regarding the amount of available NH_2_OH (**7**). For the formation of C, NH_2_OH (**7**) needs to be present in large amounts, while for the formation of U, 1 eq. of NH_2_OH (**7**) is sufficient.

This highly linked HNO_2_ (**1**) /NH_2_OH (**7**) based chemistry allows us to envision a prebiotic scenario for the formation of the nucleosides from **12** (Figure 3c). If serine aminonitrile (**12**), present in a shallow pond, is first exposed to HNO_2_ (**1**) formed by lightning in a slightly reducing CO_2_‐dominated atmosphere in the absence of any volcanic activity, a mixture of **17** and **18** is generated. If some volcanic activity resumes, allowing HNO_2_ (**1**) to convert to NH_2_OH (**7**) through reaction with volcanic SO_2_, a mixture of **21**, either with **19** or with **22** will be generated. Which mixture is finally formed, depends on the pH and the amount of NH_2_OH (**7**). At slightly acidic conditions (pH ≈ 4) a mixture of **19** and **21** is generated, while at pH 9, the mixture will contain **21** with **22**. If we imagine a two‐pond model, cyclization of **22** to 3‐aminoisoxazole **25** (towards C) can happen in one pond in the presence of SO_2_ (pH ≈ 2–3). This will destroy 5‐aminoisoxazole **21**. The reaction towards U (and also G and A) has therefore to occur in a separate pond under more pH‐neutral conditions (pH ≈ 5–9).

## Discussion

Here, we discovered that direct precursors to amino acids such as serine, glycine and to all four canonical nucleosides, needed to establish the genetic code, can form from the ancestor molecule **12**, which robustly forms under prebiotically plausible conditions on the Hadean Earth in a weakly reducing CO_2_, N_2_, H_2_O, SO_2_ dominated atmosphere.^[^ ^7^, ^8^, ^9^, ^10^ ^]^ Most importantly, our scenario shows that the emergence of life is possible without a H_2_‐atmosphere. Since such an atmosphere is only stable for a short period of time, our new scenario loosens the time and space constraints imposed by an invoked temporary and local shift of the atmosphere into a strongly reducing H_2_‐regime. Thus, it offers a more robust explanation for the emergence of life during the late Hadean. Such a weakly reducing atmosphere was so far considered to be chemically too inert to allow the formation of feedstock molecules.^[^ ^24^, ^26^ ^]^ The starting point of our pathway is the idea that UV irradiation creates formaldehyde (**2**, 3 Mt/y),^[^ ^38^, ^39^ ^]^ which produces glycolaldehyde (**3**) together with formic acid (**5**). This idea recently gained strong experimental support.^[^ ^17^ ^]^

Lightning through such an atmosphere generates NO (0.5–0.7 Mt/y)^[^ ^16^, ^33^, ^34^, ^35^, ^36^, ^37^ ^]^ and this NO gives in water ultimately NO_2_^−^ (**1**) and NO_3_^−^. NO_2_^−^ (**1**) can also form from NO_3_^−^ along published pathways.^[^ ^36^, ^37^ ^]^ Volcanic eruptions furnish SO_2_,^[^ ^11^, ^12^, ^13^ ^]^ which reacts with HNO_2_ (**1**) to give NH_2_OH (**7**) and NH_3_ (**8**). The concentrations of NH_2_OH (**7**), NH_3_ (**8**), and NO_2_^−^, as well as the pH are *grosso modo* controlled by the amount of SO_2_ and hence the level of volcanic activity.

We show that under these conditions, **2** plus **3** plus **7** in the presence of ammonia generates amino acid precursors such as Gly and Ser aminonitriles. Along the way, HCN is formed allowing parallel Strecker chemistry.^[^ ^62^ ^]^ Most importantly, we show that the serine aminonitrile **12** plus **1** gives cyanoacetaldehyde (**17**), which is converted with NO_2_^−^ or NH_2_OH (**7**) to the oxime‐, amidoxime‐, and nitrile‐containing feedstock molecules **19**, **22**, and **24**, which function as direct precursors for the canonical nucleosides.

**Figure 4:**
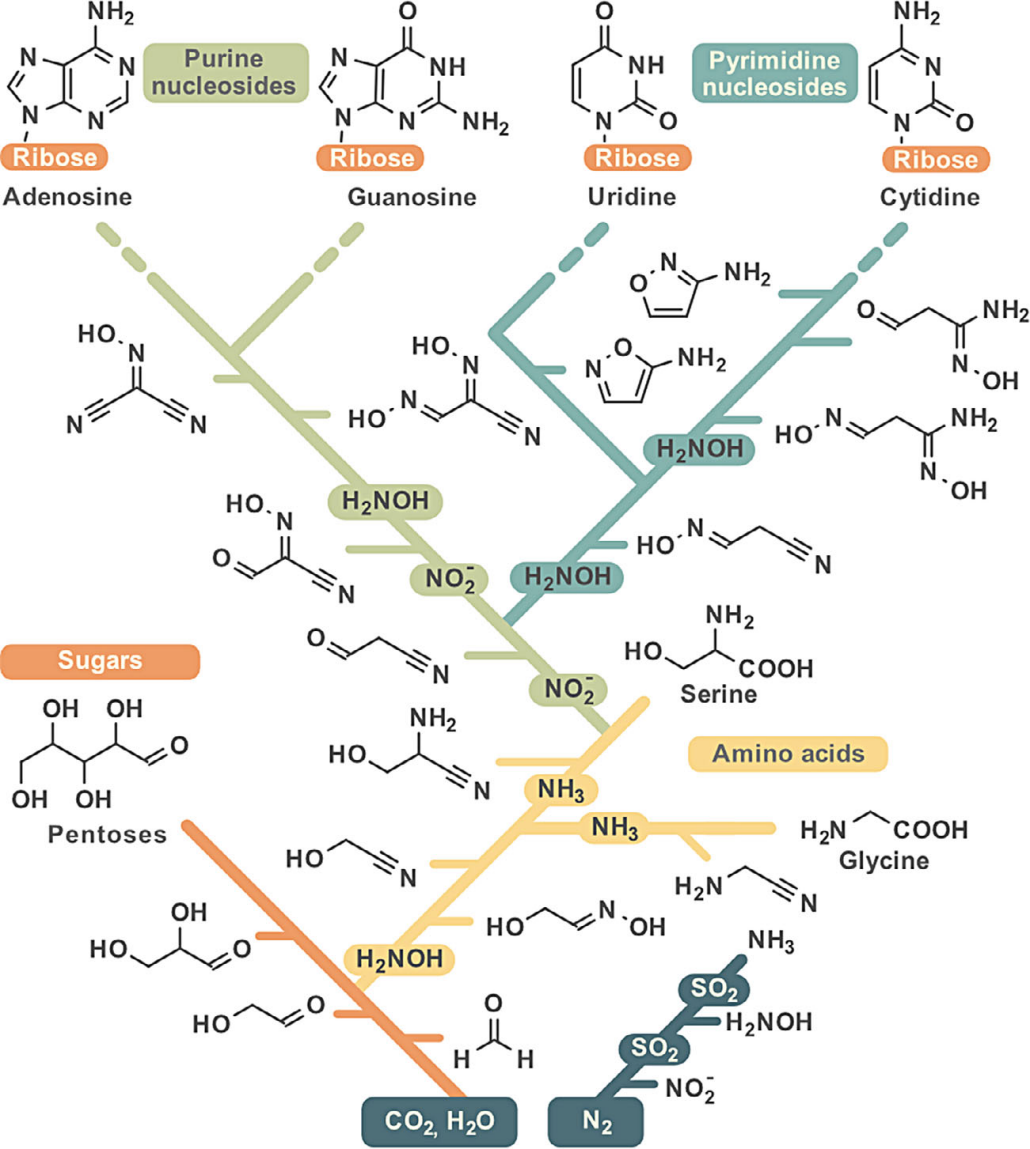
“Phylochemical tree” of the building blocks of life. Bold lines represent chemical pathways leading to the main biomolecules. Different colors correspond to distinct paths leading to various classes of final compounds. Thin lines indicate the positions of intermediate compounds within the pathways. Ribose = Ribosides, which are a mixture of α,β‐ribofuranosides and α,β‐ribopyranosides.

The discovery that the essential biomolecules can all be traced back to the common chemical ancestor **12** allows us to formulate a “phylochemical tree” of organics (Figure 4). The formation of sugars from glycolaldehyde **3** and glyceraldehyde **4** requires no N‐fixation step. These carbonyl compounds are at the root of the tree. Formation of aminonitriles such as **12** requires two N‐incorporation steps with first hydroxylamine (**7**) and second NH_3_ (**8**).

The availability of SO_2_ governs the reaction pathways taken by the serine aminonitrile (**12**). Formation of pyrimidines needs four to five N‐fixation steps in total. The first with NH_2_OH (**7**), the second with NH_3_ (**8**) to create **12**, the third with HNO_2_ (**1**) and finally with one or two molecules of NH_2_OH (**7**). Towards the purines in total five N‐fixation steps are required. Again, NH_2_OH (**7**), and NH_3_ (**8**) are required to generate **12**, which is next converted with two molecules of HNO_2_ (**1**) and finally with one molecule of NH_2_OH (**7**) to the central purine precursor **20**.

## Conclusion

We show that the whole chemistry proceeds in a temperature regime between 25 and 70 °C and in pH environments between 2 and 9. This is all prebiotically plausible. We show that these pH‐shifts are realistically achievable with either ammonia or sulfur dioxide. The higher temperatures are locally available through volcanic activity. The chemical model described here provides a comprehensive pathway from inorganic matter (CO_2_, NO) to the molecules of life. As life is not possible without compartmentalization, we find currently a scenario most realistic with a system of connected ponds, with each pond containing only a small number of different molecules. The ponds may have existed in different environments either closer or further away from volcanic activities. Flooding and tectonic moves may have connected these different ponds at some point in time to allow mixing of the contents. Dry‐down steps have certainly allowed concentrating of reactants such as formic acid. Despite these constraints, the presented chemistry is simple and based on just a small set of key starting molecules such as formaldehyde, glycolaldehyde, and hydroxylamine.

With the fact that billions of rocky exoplanets are present in the Universe, with many of them likely surrounded by a CO_2_‐dominated (weakly reducing) atmosphere,^[^ ^32^, ^63^, ^64^, ^65^ ^]^ the here described chemistry suggests that the molecules of life can robustly form at many places in the universe.

## Supporting Information

The authors have cited additional references within the Supporting Information.^[^ ^66^, ^67^, ^68^, ^69^ ^]^ Additional data is publicly available on Figshare. (https://doi.org/10.6084/m9.figshare.28202876.v2).

## Conflict of Interests

The authors declare no conflict of interest.

## Supporting information



Supporting Information

## Data Availability

The data that support the findings of this study are openly available in Figshare at https://doi.org/10.6084/m9.figshare.28202876.v2, reference number 28202876.
