# Supplementary material for: The Origin of the Feedstock Molecules for Life on the Hadean Earth
Source: Angew Chem Int Ed Engl. 2025 Sep 1;64(42):e202512374. doi: 10.1002/anie.202512374 (PMC12518691; doi:10.1002/anie.202512374)
Supplement: Supplementary file 1 — Supporting Information [file ANIE-64-e202512374-s001.docx]

Supplementary Materials for

**Title: The Origin of the Feedstock Molecules for Life on the Hadean Earth**

**Authors:** Nikolai Diukarev^1^†, Erik Boinowitz^1^†, Jonas Feldmann^1^†, Stefan Wiedemann^1^, Aleksandr Mikheev^1^, Sidney Becker^1^ and Thomas Carell^1^*

**Affiliations:**

^1^Institute for Chemical Epigenetics, Department of Chemistry, Ludwig-Maximilians-Universität München; Würmtalstrasse 201, 81375 Munich, Germany

* Corresponding author. Tel.: 0049 (0)89 218077755. Email: [Thomas.carell@lmu.de](mailto:Thomas.carell@lmu.de). Web: [www.carellgroup.de](http://www.carellgroup.de)

†These authors contributed equally to this work

**The PDF file includes:**

Materials and Methods

Supplementary Text

Figs. S1 to S17

Tables S1 to S2

**Other Supplementary Materials for this manuscript include the following:**

Data S2 to S18

**Materials and Methods**

Chemicals and solvents were purchased from Sigma-Aldrich, Fluka, BLDpharm, ABCR, Carbosynth, TCI or Acros organics and used without further purification.

^1^H- and ^13^C-NMR spectra were recorded on a Bruker Ascend^TM^ 500 spectrometer and calibrated to the residual solvent peak. Multiplicities are abbreviated as follows: s = singlet, d = doublet, t = triplet, q = quartet, m = multiplet, br = broad. Quantitative NMR was carried out using maleic acid or dimethyl sulfone as internal standards. Yields of prebiotic reactions were calculated by analyzing a sample of the reaction mixture by ^1^H-NMR, integrating product peaks against the signal of the standard and extrapolating to the total volume of the reaction. Prebiotic reactions were carried out in triplicate and the yields calculated as a range from lowest to highest.

A Thermo Scientific Vanquish system coupled to a Thermo Scientific QExactive HF mass spectrometer was used for the high-resolution mass spectrometry (HRMS) analysis of hydroxycarbonimidoyl dicyanide **20** synthesis and for characterization of compounds. Ionization was done by a HESI source and ions were scanned in the negative polarity mode over a full-scan range of m/z 50–500 with a resolution of 120,000, an AGC target of 3x10^6^ and a maximum IT of 200 ms. HESI Tune parameters are: Capillary temperature 320 °C, sheath gas flow rate 20 au, aux gas flow rate 4 au, sweep gas flow rate 0 au, spray voltage 3.5 kV, S-lens RF level 50, aux gas temperature 55 °C. Compounds were separated on an Interchim Uptisphere 120 Å column (HDO-C18, 3 µm, 2.1 × 150 mm) at 30 °C. Elution buffers were 5 mm NH_4_OAc aqueous buffer A, brought to pH 4.9 with glacial acetic acid (200 μL/L) and an organic buffer B of 2 mm NH_4_COOH in 80 % acetonitrile (Roth, Ultra LC-MS grade, purity ≥99.98) with a flow rate of 0.20 mL/min.

The gradient started at 100 % solvent A for 2 min, followed by an increase of solvent B to 7.5 % over 15 min. From 15 min to 18 min, solvent B was increased to 100 % and maintained at this amount for 5 min before returning to 100 % solvent A in 1 min and a 3 min re-equilibration period at 100 % solvent A. For each measurement 10 µL of sample were subjected to HRMS. The ion chromatograms were extracted from the total ion current (TIC) chromatogram, and the areas under the curves were integrated.

IR measurements were performed on Perkin Elmer Spectrum BX FT-IR spectrometer with a diamond-ATR (Attenuated Total Reflection) setup.

The X-ray intensity data were measured on a Bruker D8 Venture TXS system equipped with a multilayer mirror monochromator and a Mo Kα rotating anode X-ray tube (λ = 0.71073 Å). The frames were integrated with the Bruker SAINT software package.^(^*^64^*^)^ Data were corrected for absorption effects using the Multi-Scan method (SADABS).^(^*^65^*^)^ The structure was solved and refined using the Bruker SHELXTL Software Package.^(66)^ All C-bound hydrogen atoms have been calculated in ideal geometry riding on their parent atoms while the N-bound hydrogen atoms have been refined freely. The figures have been drawn at the 50% ellipsoid probability level.

**Fundamental Reaction Pathways**

**S1.1**. Formation of NO: ^(^*^33, 35^*^)^

| CO_2_ + hv → O + CO | (1) |
| --- | --- |
| N_2_ + O → NO + N | (2) |
| N + CO_2_ → NO + CO | (3) |

**S1.2**. Formation of NO_x_^-^:^(34, 35)^

| H_2_O + hv → H + OH | (4) |
| --- | --- |
| CO + H + M → HCO + M | (5) |
| HCO + NO → HNO + CO | (6) |
| HNO → H^+^ + NO^-^ | (7) |
| NO^-^ + NO → N_2_O_2_^-^ | (8) |
| N_2_O_2_^-^ + NO → N_3_O_3_^-^ | (9) |
| N_x_O_x_^-^ → NO_3_^-^ + NO_2_^-^(**1**) + N_2_O | (10) |

**S.1.3.** Formation of H_2_CO (**2**):^(38)^

| CO_2_ + hv → CO + O | (11) |
| --- | --- |
| H_2_O + hv → H + OH | (12) |
| H + CO + M → HCO + M | (13) |
| HCO + HCO → H_2_CO (**2**) + CO | (14) |

**S.1.4.** Formation of NH_2_OH (**7**):^(46)^

| NO_2_^-^ + 2 HSO_3_^-^ → HON(SO_3_^-^)_2_ (**6**) | (15) |
| --- | --- |
| HON(SO_3_^-^)_2_ (**6**) + 2 H_2_O → NH_2_OH (**7**) + 2 HSO_4_^-^ | (16) |

**S.1.5.** Formation of NH_3_ (**8**):^(15, 67)^

| HON(SO_3_^-^)_2_ (**6**) + SO_2_ → HN(SO_3_^-^)_2_ + SO_3_ | (17) |
| --- | --- |
| HN(SO_3_^-^)_2_ + 2 H_2_O → NH_3_ (**8**) + 2 HSO_4_^-^  H_2_NOH (**7**) + SO_2_ → NH_3_ (**8**) + SO_3_  NO_2_^-^ (**1**) + 6 Fe^2+^ + 7 H^+^ → 6 Fe^3+^ + 2 H_2_O + NH_3_ (**8**) | (18)  (19)  (20) |

**S.1.6.** Formation of formic acid (**5**), guanidinium (**15**) and urea (**16**):^(20, 47, 50-53, 55)^

| H_2_CO (**2**) + NH_2_OH (**7**) → CH_2_NOH (**14**) + H_2_O | (21) |
| --- | --- |
| CH_2_NOH (**14**) → HCN (**13**) + H_2_O | (22) |
| HCN (**13**) + 2 H_2_O → HCOOH (**5**) + NH_3_ (**8**)  HCN (**13**) → H_2_NCN | (23)  (24) |
| H_2_NCN + NH_3_ (**8**) → HNC(NH_2_)_2_ (**15**) | (25) |
| H_2_NCN + H_2_O → CO(NH_2_)_2_ (**16**) | (26) |
| CO(NH_2_)_2_ (**16**) → HOCN + NH_3_ (**8**) | (27) |

**Abiotic nitrogen fixation**

**Hydroxylammonium hydrogensulfite** **(7·HSO_3_)**

**S2.** Prebiotic formation:

Sodium nitrite (**1·Na**, 0.690 g, 10 mmol, 1.0 eq.) was dissolved in water (500 mL), resulting in an initial 20 mm of concentration of nitrite (**1**). After letting dry down for one week at 35°C, the volume had reduced to 40 mL. Sodium bisulfite (0.697 g, 6.7mmol, 1.0 eq.) was added and sulfur dioxide was generated *in situ* by dropping an aqueous solution of HCl (6 m) onto sodium bisulfite and bubbled into the solution above until a pH of 3 was reached. The flow of sulfur dioxide was stopped, and the solution was incubated at 70°C for 3 hours. The solvent was removed by lyophilization to yield hydroxylammonium hydrogensulfite (**7·HSO_3_**) in 27-37% yield and ammonium hydrogensulfite in 15-32% as a crystalline solid as determined by quantitative ^1^H-NMR with dimethyl sulfone as an internal standard. The presence of **7·HSO_3_** was confirmed by spiking with commercially available hydroxylammonium hydrochloride (Fig. S1).

**^1^H-NMR** (500 MHz, DMSO-*d_6_*) *δ* = 10.20 (s, 3H, NH), 9.99 (br ,1H, OH) ppm.


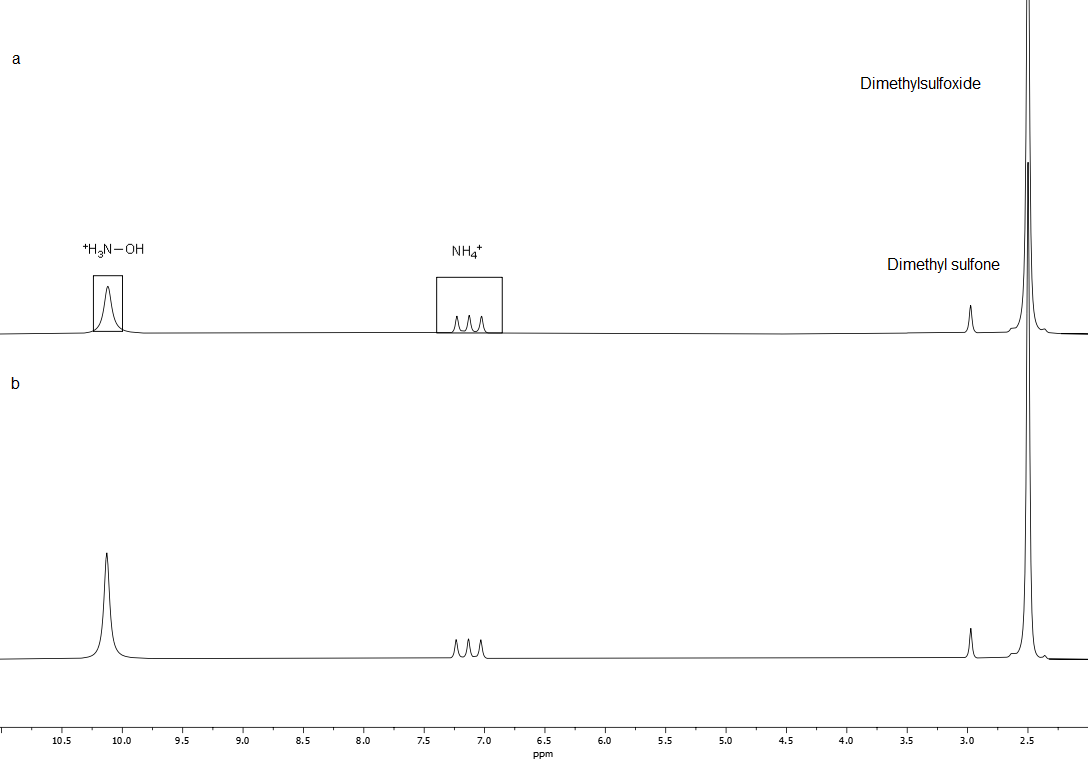


**Fig. S1.** ^1^H-NMR spectra (500 MHz, DMSO-d_6_, 2.0–11.0 ppm) of prebiotically produced hydroxylammonium hydrogensulfite (**7·HSO_3_**) from nitrite (**1**). (a) Reaction mixture after treating with sulfur dioxide for 3 hours at 70°C and pH 3 (b) reaction mixture spiked with commercially available hydroxylammonium chloride.

**Ammonium hydrogensulfite (8·HSO_3_)**

**S3.** Prebiotic formation:

Sodium nitrite (**1·Na**, 0.690 g, 10 mmol, 1.0 eq.) was dissolved in water (500 mL), resulting in an initial 20 mm of concentration of nitrite (**1**). After letting dry down for one week at 35°C, the volume had reduced to 40 mL. Sodium bisulfite (0.697 g, 6.7mmol, 0.67 eq.) was added and sulfur dioxide was generated in situ by dropping an aqueous solution of HCl (6 m) onto sodium bisulfite and bubbled into the solution above until a pH of 2 was reached. The flow of sulfur dioxide was stopped, and the solution was incubated at 70°C for 3 hours. The solvent was removed by lyophilization to yield ammonium hydrogensulfite (**8·HSO_3_)** in 26-42% yield and hydroxylammonium hydrogensulfite in 13-19% yield as crystalline solids as determined by quantitative ^1^H-NMR using dimethyl sulfone as an internal standard. The presence of **8·HSO_3_** was confirmed by spiking with commercially available ammonium hydrochloride (Fig. S2).

**^1^H-NMR** (500 MHz, DMSO-*d_6_*) *δ* = 7.17 (t, *J* = 50.9 Hz, 4H, NH) ppm.


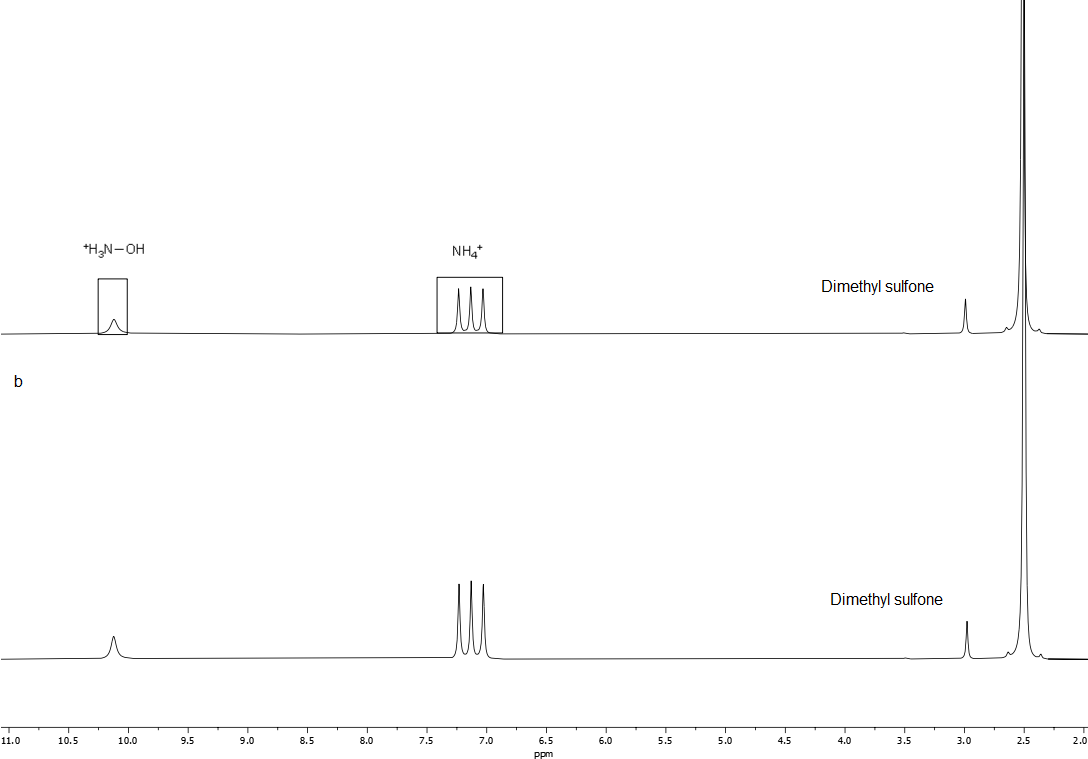


**Fig. S2. ^1^H-NMR spectra (500 MHz, DMSO-d_6_, 2.0–11.0 ppm) of prebiotically produced ammonium hydrogensulfite (8·HSO_3_) from nitrite (1).** (a) Reaction mixture after treating with sulfur dioxide for 3 hours at 70°C and pH 2 (b) reaction mixture spiked with commercially available ammonium chloride.

**Amino Acids**

**Sodium 1,2-dihydroxyethane-1-sulfonate (3@B)**

1,4-Dioxan-2,5-diol (300 mg, 2.50 mmol, 0.5 eq.) was dissolved in H_2_O (1.0 mL) and 2m HCl (100 µL). A solution of NaHSO_3_ (520 mg, 5.00 mmol, 1.0 eq.) in H_2_O (2.5 mL) was added dropwise and the reaction mixture was stirred for 30 min at room temperature. MeCN (10 mL) was added and formed precipitates were filtered off. The solvents were removed *in vacuo*. The residue was taken up in MeOH, filtered, the solvent removed *in vacuo* and the residue dried under high vacuum. The product **3@B** was obtained as a white solid (720 mg, 4.39 mmol, 88%).

**^1^H-NMR** (500 MHz, DMSO-*d_6_*) *δ* = 5.53 (d, *J* = 6.1 Hz, 1H, SO_3_CHOH), 4.36 (t, *J* = 6.0 Hz, 1H, CH_2_OH), 3.92 (dt, *J* = 6.1, 4.1 Hz, 1H, SO_3_CH), 3.65 (ddd, *J* = 11.4, 6.0, 4.1 Hz, 1H, CH_2_a), 3.41 (ddd, *J* = 11.4, 6.0, 4.1 Hz, 1H, CH_2_b) ppm. **^13^C-NMR** (126 MHz, DMSO-*d_6_*)*δ* = 83.45 (SO_3_CH), 62.52 (CH_2_) ppm. **IR** (cm^-1^): 𝜈̃ = 3331 (w), 2359 (vw), 1343 (w), 1263 (m), 1178 (s), 1132 (m), 1079 (s), 1045 (vs), 989 (m), 894 (m), 703 (s). **HRMS** (ESI^-^): calc.: [C_2_H_5_O_5_S]^-^ 140.9863, found: 140.9862.

**Sodium 1,2,3-trihydroxypropane-1-sulfonate (4@B)**

3,6-Dihydroxy-1,4-dioxan-2,5-dimethanol (450 mg, 2.5 mmol, 0.5 eq.) was dissolved in H_2_O (1.0 mL) and 2 m HCl (100 µL). A solution of NaHSO_3_ (520 mg, 5.00 mmol, 1.0 eq.) in H_2_O (2.5 mL) was added dropwise and the reaction mixture was stirred for 20 min at room temperature. MeCN (10 mL) was added and formed precipitates were filtered off. The solvents were removed *in vacuo*. The residue was taken up in MeOH, filtered and the precipitate dried under high vacuum. The product **4@B** was obtained as a colorless oil (quantitative yield).

**^1^H-NMR** (500 MHz, DMSO-*d_6_*) *δ* = 5.59 (d, *J* = 6.8 Hz, 1H, SO_3_CHOH), 4.88 (d, *J* = 2.1 Hz, 1H, CHOH), 4.30 (dd, *J* = 7.0, 5.2 Hz, 1H, CH_2_OH), 3.86 (t, *J* = 6.8 Hz, 1H, SO_3_CH), 3.61-3.57 (m, 2H, CH_,_ CH_2_a), 3.41-3.35 (m, 1H, CH_2_b) ppm. **^13^C-NMR** (126 MHz, DMSO-*d_6_*)*δ* = 82.84 (SO_3_CH), 71.91 (CH), 62.24 (CH_2_) ppm. **IR** (cm^-1^): 𝜈̃ = 3375 (m), 2961 (vw), 1643 (vw), 1409 (w), 1174 (vs), 1068 (m), 1029 (vs), 703 (m). **HRMS** (ESI^-^): calc.: [C_3_H_7_O_6_S]^-^ 170.9969, found: 170.9968.

**2-Hydroxyacetaldehyde oxime (9)**

Synthetic reference:

In a 50 mL Falcon-tube equipped with a stirring bar, 1,4-dioxane-2,5-diol (1.20 g, 10.0 mmol, 0.5 eq.) was dissolved in water (7.55 mL). After adding hydroxylamine (**7**, 50 wt% in H_2_O; 2.64 g, 2.45 mL, 40.0 mmol, 2.0 eq.), the white suspension was stirred for 1 hour at room temperature. After the clear solution was lyophilized, 2-hydroxyacetaldehyde oxime (**9**) was obtained as a colorless oil (0.99 g, 13.2 mmol, 66%).

NMR analysis revealed an isomeric ratio of 62 (*Z*) : 38 (*E*).

**^1^H-NMR** (500 MHz, DMSO_2_-*d_6_*) *δ* = 10.90 (br, *E*, 1H, HCNOH), 10.65 (br, *Z*, 1H, HCNOH), 7.32 (t, *Z*, *J* = 5.8 Hz, 1H, CH), 6.71 (t, *E*, *J* = 3.8 Hz, 1H, CH), 4.99 (br, *Z*, 1H, OH), 4.19 (d, *E*, *J* = 3.8 Hz, 2H, CH_2_), 3.96 (d, *Z*, *J* = 5.8 Hz, CH_2_), 3.47 (br, *E,* 1H, OH) ppm. **^13^C-NMR** (126 MHz, DMSO-*d_6_*) *δ =* 152.9 (*E*, CH), 149.4 (*Z*, CH), 58.8 (*Z*, CH_2_), 55.8 (*E*, CH_2_) ppm. **IR** (cm^-1^): 𝜈̃ = 3243 (s), 2882 (w), 1635 (w), 1447 (m), 1282 (m), 1224 (w), 1104 (w), 1046 (s), 989 (vs), 931 (vs), 690 (vs). **HRMS** (ESI^+^) calc.: [C_2_H_5_NO_2_]^+^ 76.0354, found: 76.0391

**S4**. Prebiotic formation from glycolaldehyde (**3**):

Glycolaldehyde (**3**, 100 µmol, 6.0 mg, 1.0 eq.) was dissolved in H_2_O (200 µL). A formic acid buffer (100 µL, 5 m HCOOH (**5**), 5 m HCOONa (**5·Na**), pH 4) and a solution of hydroxylamine (**7**, 120 µL, 120 µmol; 1.2 eq.) were added, resulting in a 240 mm concentration of glycolaldehyde (**3**). The reaction mixture was shaken at 50 °C and 750 rpm for 3 h in an Eppendorf ThermoMixer®. A sample (50 µL) of the reaction mixture in D_2_O (500 µL) was analyzed by NMR spectroscopy using dimethyl sulfone as an internal standard and spiked with synthetically obtained product. The yield of 2-hydroxyacetaldehyde oxime (**9**) was found to be quantitative.

NMR analysis revealed an isomeric ratio of 68 (*Z*): 32 (*E*).

**^1^H-NMR** (500 MHz, D_2_O) *δ* = 7.58 (t, *Z*, *J* = 5.1 Hz, 1H, CH), 6.98 (t, *E*, *J* = 3.9 Hz, 1H, CH), 4.45 (d, E, *J* = 3.9 Hz, 2H, CH_2_), 4.21 (d, Z, *J* = 5.1 Hz, CH_2_) ppm.

**S5.** Prebiotic formation from sulfonate **3@B**:

The sulfonate **3@B** (50 µmol, 8.2 mg, 1.0 eq.) was dissolved in H_2_O (150 µL). A formic acid buffer (100 µL, 5 m HCOOH (**5**), 5 m HCOONa (**5·Na)**, pH 4) and a solution of hydroxylamine (60 µL, 60 µmol; 1.2 eq.) were added, resulting in a 160 mm concentration of **3@B**. The reaction mixture was shaken at 50 °C and 750 rpm for 3 h in an Eppendorf ThermoMixer®. A sample (50 µL) of the reaction mixture in D2O (500 µL) was analyzed by NMR spectroscopy using maleic acid as an internal standard and spiked with synthetically obtained product (Fig. S3). The yield of 2-hydroxyacetaldehyde oxime (**9)** was found to be 52-56%.

NMR analysis revealed an isomeric ratio of 70 (*Z*) : 30 (*E*).


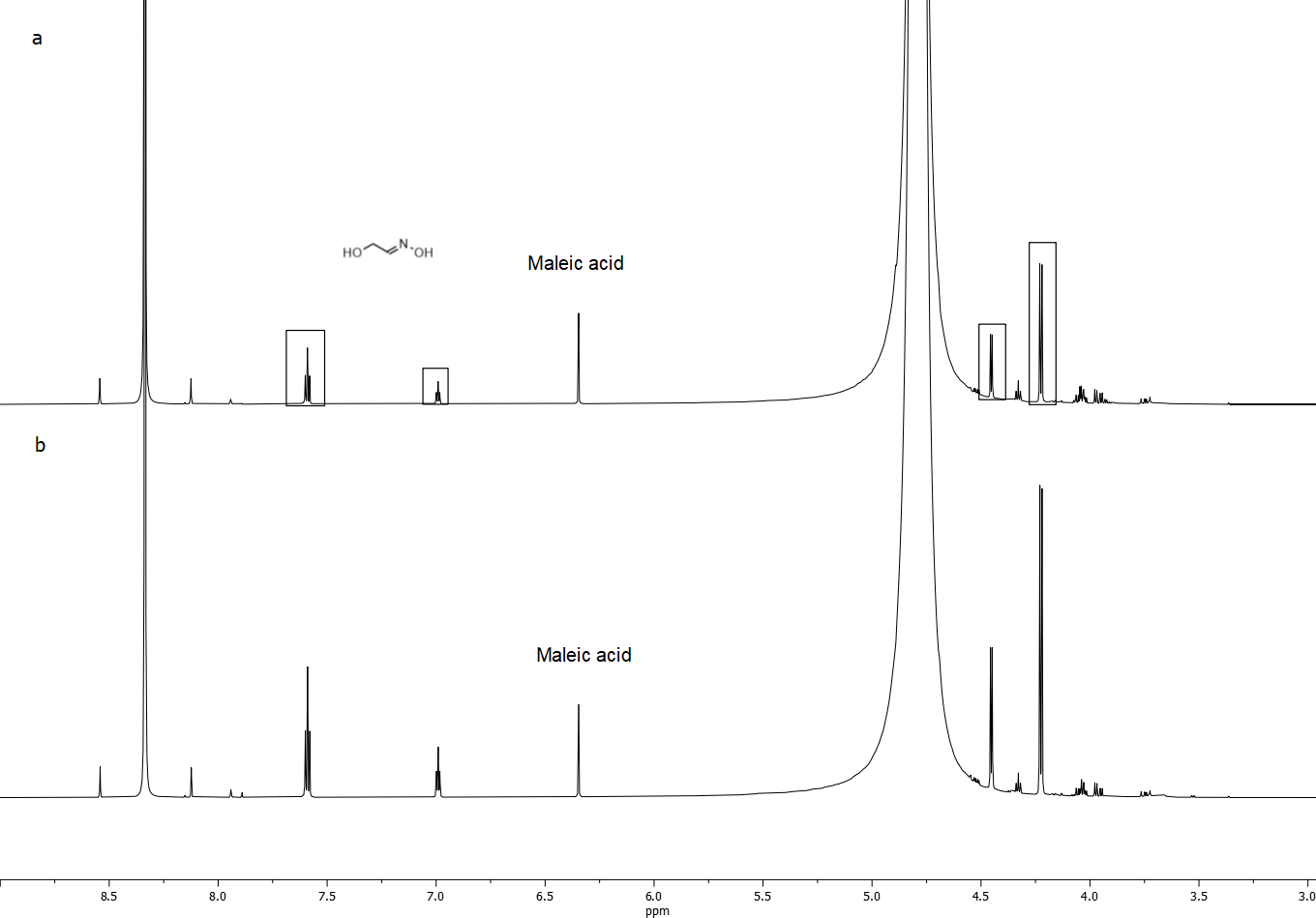


**Fig. S3.** ^1^H-NMR spectra (500 MHz, D_2_O, 3.0–9.0 ppm) of prebiotically produced 2-hydroxacetaldehyde oxime (**9**) from sulfonate **3@B**. (a) Reaction mixture after treating with hydroxylamine for 3 hours at 50°C and pH 4 (b) reaction mixture spiked with synthetically obtained **9**.

**S6.** Competition between sulfonate **3@B** and ribose for hydroxylamine (**7**):

The sulfonate **3@B** (100 µmol, 16.4 mg, 1.0 eq.) and ribose (100 µmol 15.0 mg, 1.0 eq.) were dissolved in H_2_O (200 µL). A formic acid buffer (100 µL, 5 m HCOOH (**5**), 5 m HCOONa (**5·Na**), pH 4) and a solution of either 0.6 eq. (60 µL, 60 µmol) or 1.0 eq. (100 µL, 100 µmol) hydroxylamine (**7)** were added. The reaction mixture was shaken at 25 °C and 750 rpm overnight in an Eppendorf ThermoMixer®. A sample (50 µL) of the reaction mixture in D_2_O (500 µL) was analyzed by NMR spectroscopy using dimethyl sulfone as an internal standard and spiked with synthetically obtained **9**. With 0.6 eq. of hydroxylamine (**7**), exclusive formation of oxime **9** was observed (Fig. S4), whereas with 1.0 eq. of **7**, only minor reaction between ribose and hydroxylamine was observed (Fig. S5).


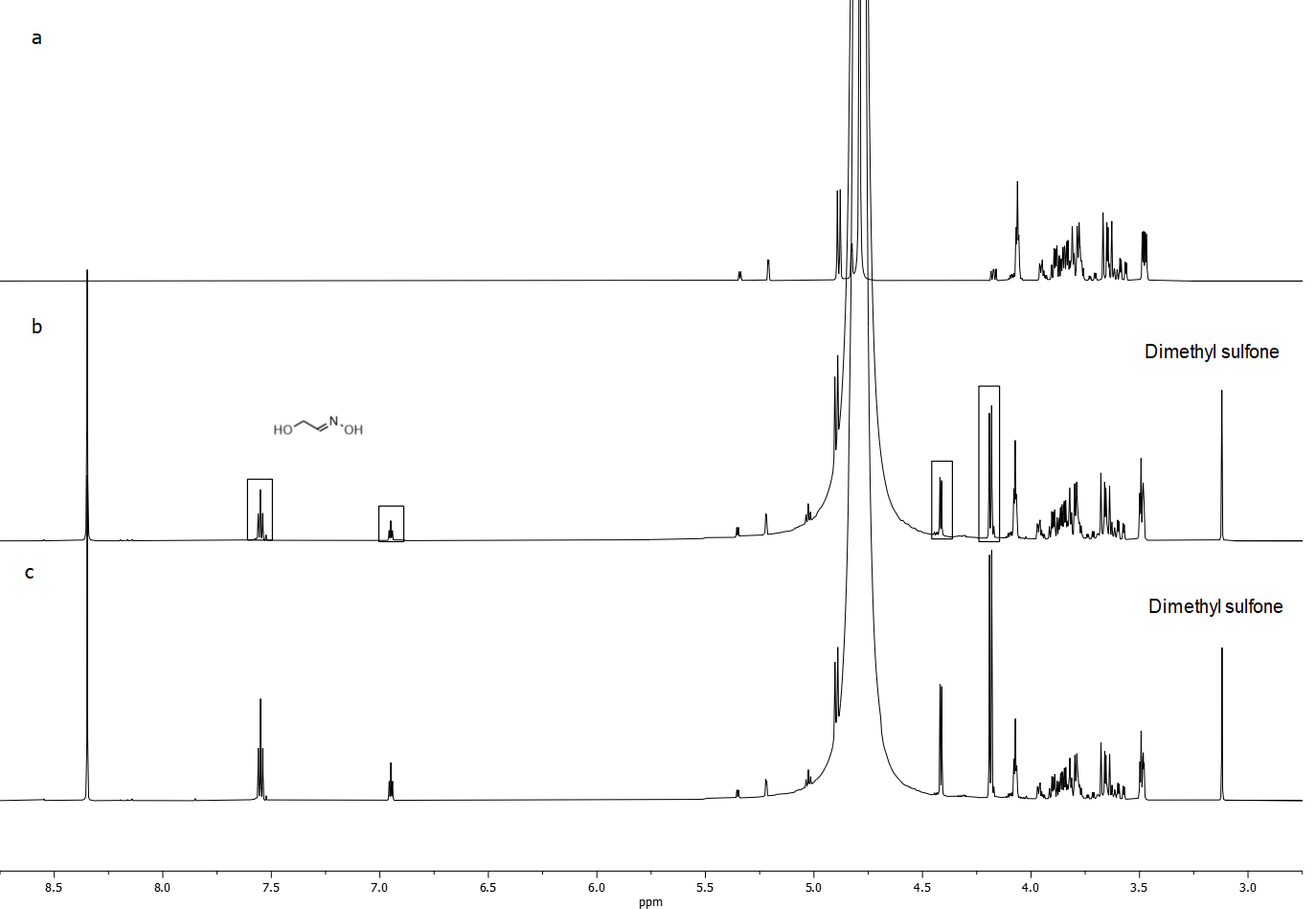


**Fig. S4: ^1^H-NMR spectra (500 MHz, D_2_O, 2.8–8.8 ppm) of the reaction between ribose, sulfonate 3@B and hydroxylamine (7, 0.6 eq.).** (a) Ribose (b) reaction mixture after treatment with hydroxylamine at 25°C and pH 4 overnight (c) reaction mixture spiked with synthetically obtained **9**. Reaction between ribose and hydroxylamine (**7**) could not be observed.


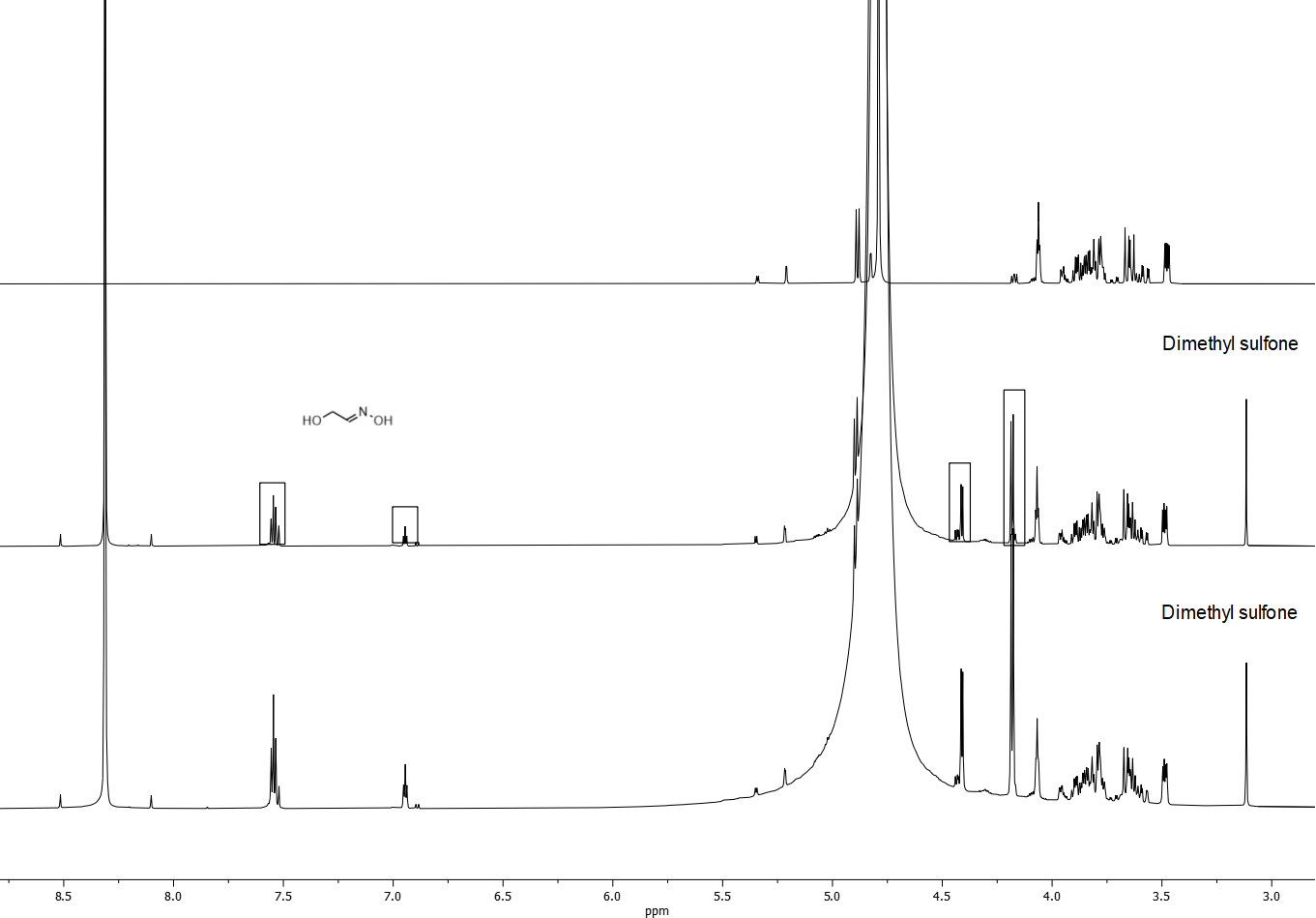


**Fig. S5:** ^1^H-NMR spectra (500 MHz, D_2_O, 2.8–8.8 ppm) of the reaction between ribose, sulfonate **3@B** and hydroxylamine (**7**, 1.0 eq.). (a) Ribose (b) reaction mixture after treatment with hydroxylamine at 25°C and pH 4 overnight (c) reaction mixture spiked with synthetically obtained **9**. Only minor reaction between ribose and hydroxylamine (**7**) could be observed.

*.*

**Glycolonitrile (10)**

**S7:** Prebiotic formation from glycolaldehyde **3** with 1 m HCOOH (**5**) buffer:

Glycolaldehyde (**3**, 100 µmol, 6.0 mg, 1.0 eq.) was dissolved in H_2_O (4 mL). A formic acid buffer (1 mL, 5 m HCOOH (**5**), 5 m HCOONa (**5·Na**), pH 4) and a solution of hydroxylamine (120 µL, 120 µmol; 1.2 eq.) were added, resulting in a 20 mm concentration of glycolaldehyde (**3**) and a 1 m concentration of formic acid buffer. The reaction mixture was shaken at 50 °C and 750 rpm for 2 weeks in an Eppendorf ThermoMixer for the solvent to evaporate until a reaction volume of 500 µL was reached. A sample (50 µL) of the reaction mixture in D_2_O (500 µL) was analyzed by NMR spectroscopy using dimethyl sulfone as an internal standard and spiked with commercially available product. The yield of glycolonitrile (**10**) was found to be 21-26%.

**S8.** Prebiotic formation from glycolaldehyde (**3**) with 5 m HCOOH (**5**) buffer:

Glycolaldehyde (**3**, 100 µmol, 6.0 mg, 1.0 eq.) was dissolved in H_2_O (50 µL). A formic acid buffer (150 µL, 5 m HCOOH (**5**), 5 m HCOONa (**5·Na**), pH 4) and a solution of hydroxylamine (**7**, 120 µL, 120 µmol; 1.2 eq.) were added, resulting in a 310 mm concentration of glycolaldehyde (**3**). The reaction mixture was shaken at 50 °C and 750 rpm for 3 d in an Eppendorf ThermoMixer. A sample (50 µL) of the reaction mixture in D_2_O (500 µL) was analyzed by NMR spectroscopy using dimethyl sulfone as an internal standard and spiked with commercially available product. The yield of glycolonitrile (**10**) was found to be 34-38%.

**^1^H-NMR** (500 MHz, D_2_O) *δ* = 4.43 (s, 2H, CH_2_) ppm.

**S9.** Prebiotic formation from oxime **9**:

2-Hydroxyacetaldehyde oxime (**9**, 50 µmol, 3.8 mg, 1.0 eq.) was dissolved in H_2_O (50 µL). A formic acid buffer (100 µL, 5 m HCOOH (**5**), 5 m HCOONa (**5·Na**), pH 4) was added, resulting in a 330 mm concentration of **7**. The reaction mixture was shaken at 50 °C and 750 rpm for 3 d in an Eppendorf ThermoMixer®. A sample (50 µL) of the reaction mixture in D_2_O (500 µL) was analyzed by NMR spectroscopy using dimethyl sulfone as an internal standard and spiked with commercially available product (Fig. S6). The yield of hydroxyacetonitrile (**10)** was found to be 37-42%.

*
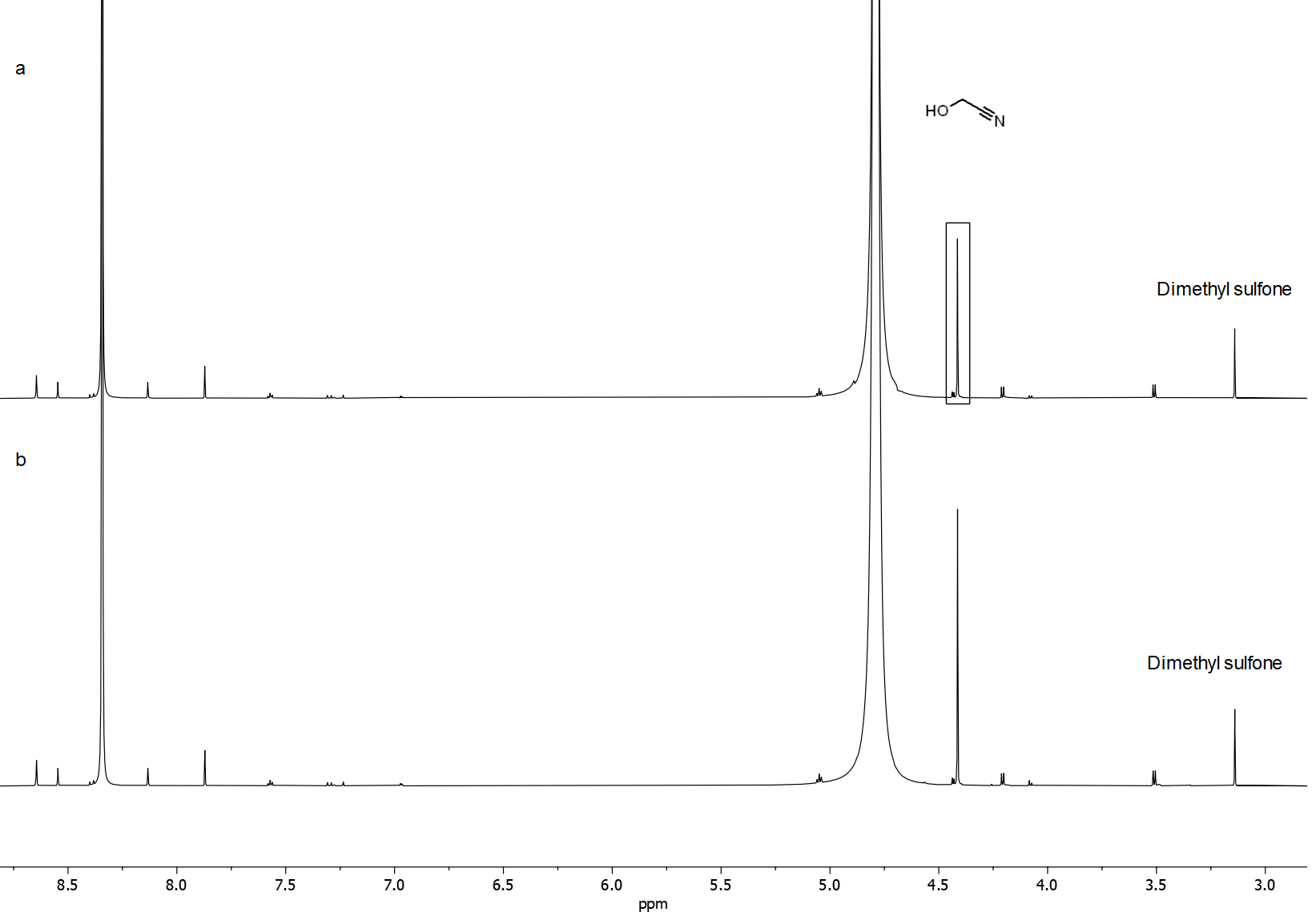
*

**Fig. S6. ^1^H-NMR spectra (500 MHz, D_2_O, 2.8–8.8 ppm) of prebiotically produced hydroxyacetonitrile (10) from oxime 9.** (a) Reaction mixture after treatment with formic acid (**5**) for 3 days at 50°C and pH 4 (b) reaction mixture spiked with commercially available **10**.

**Aminoacetonitrile (glycine aminonitrile) (11)**

**S10.** Prebiotic formation from glycolaldehyde (**3**):

Glycolaldehyde (**3**, 100 µmol, 6.0 mg, 1.0 eq.) was dissolved in H_2_O (50 µL). A formic acid buffer (150 µL, 5 m HCOOH (**5**), 5 m HCOONa (**5·Na**), pH 4) and a solution of hydroxylamine (**7**, 120 µL, 120 µmol; 1.2 eq.) were added, resulting in a 310 mm concentration of glycolaldehyde (**3**). The reaction mixture was shaken at 50 °C and 750 rpm in an Eppendorf ThermoMixer® for 3d. An ammonia buffer (225 µL, 11 eq. NH_3_ (**8)**_,_ 11 eq. NH_4_Cl (**8·Cl**), pH 9) was added and the mixture was further shaken for 1 d. A sample (100 µL) of the reaction mixture in D_2_O (500 µL) was analyzed by NMR spectroscopy using dimethyl sulfone as an internal standard and spiked with commercially available product (Fig. S7). The yield of aminoacetonitrile (**11)** was found to be 34-35%.

**^1^H-NMR** (500 MHz, D_2_O) *δ* = 3.62 (s, 2H, CH_2_) ppm.


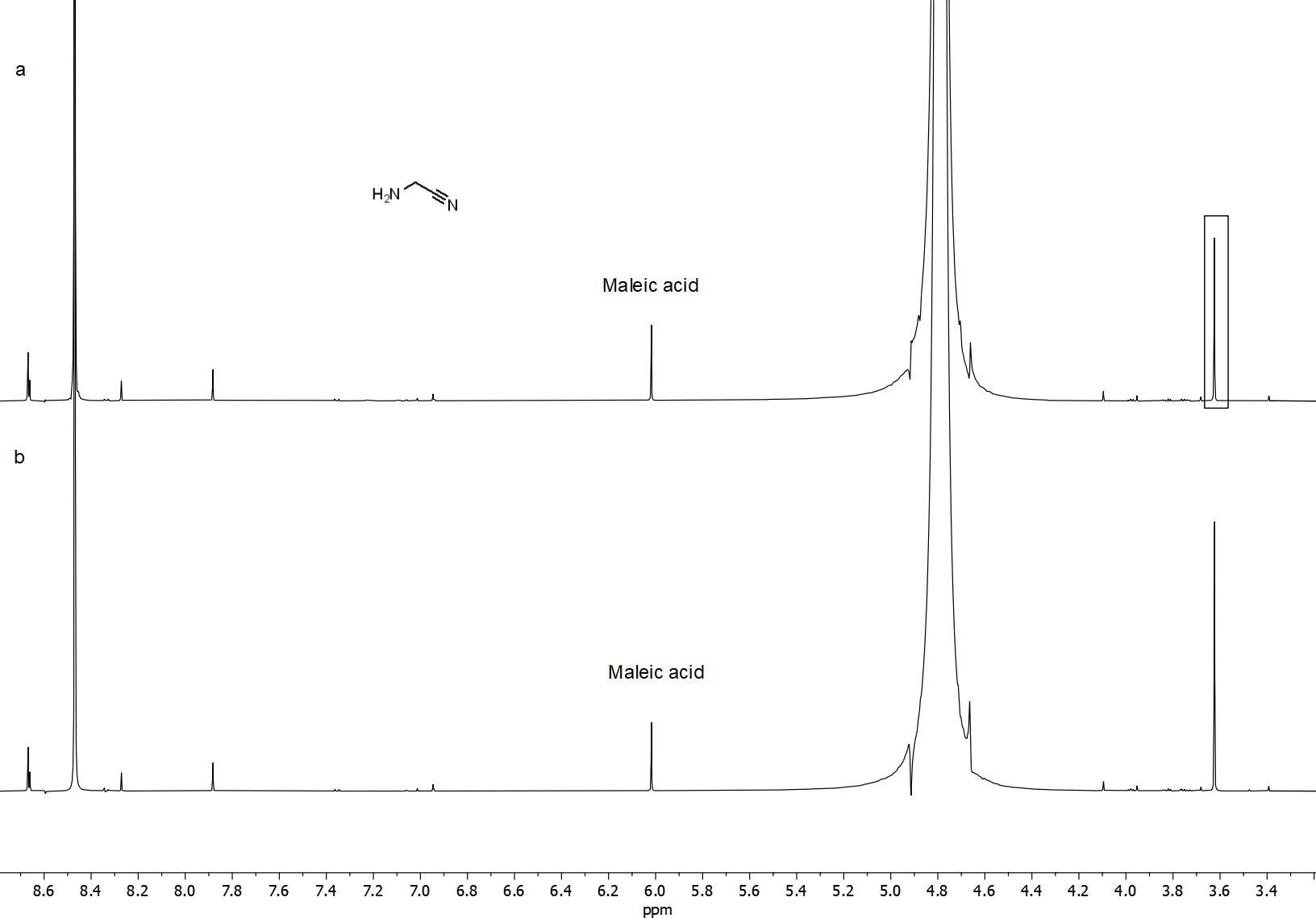


**Fig. S7. ^1^H-NMR spectra (500 MHz, D_2_O, 3.2–8.8 ppm) of prebiotically produced aminoacetonitrile 11 from glycolaldehyde (3).** (a) Reaction mixture after treatment with hydroxylamine (**7**) and formic acid (**5**) for 3 days at pH 4, 50°C and basification with ammonia (**8**) to pH 9 (b) reaction mixture spiked with commercially available **11**.

**2-Amino-3-hydroxypropanenitrile (serine aminonitrile) (12)**

**S11.** Prebiotic one-pot formation of aminonitriles **11** and **12** from glycolaldehyde **3**:

Glycolaldehyde (**3**, 100 µmol, 6.0 mg, 1.0 eq.) was dissolved in H_2_O (50 µL). A formic acid buffer (150 µL, 5 m HCOOH (**5**), 5 m HCOONa (**5·Na**), pH 4) and a solution of hydroxylamine (**7**, 60 µL, 60 µmol; 0.6 eq.) were added, resulting in a 380 mm concentration of glycolaldehyde (**3**). The reaction mixture was shaken at 50 °C and 750 rpm in an Eppendorf ThermoMixer® for 3 days. An ammonia buffer (225 µL, 11 eq. NH_3_ (**8**)_,_ 11 eq. NH_4_Cl (**8·Cl**), pH 9) was added. The mixture was further shaken for 1 d. A sample (100 µL) of the reaction mixture in D_2_O (500 µL) was analyzed by NMR spectroscopy using dimethyl sulfone as an internal standard and spiked with the commercially available products (Fig. S8). The concurrent one-pot formation of aminoacetonitrile (**11)** in 17-18% yield and 2-amino-3-hydroxypropanenitrile (**12)** in 16-17% yield was observed.


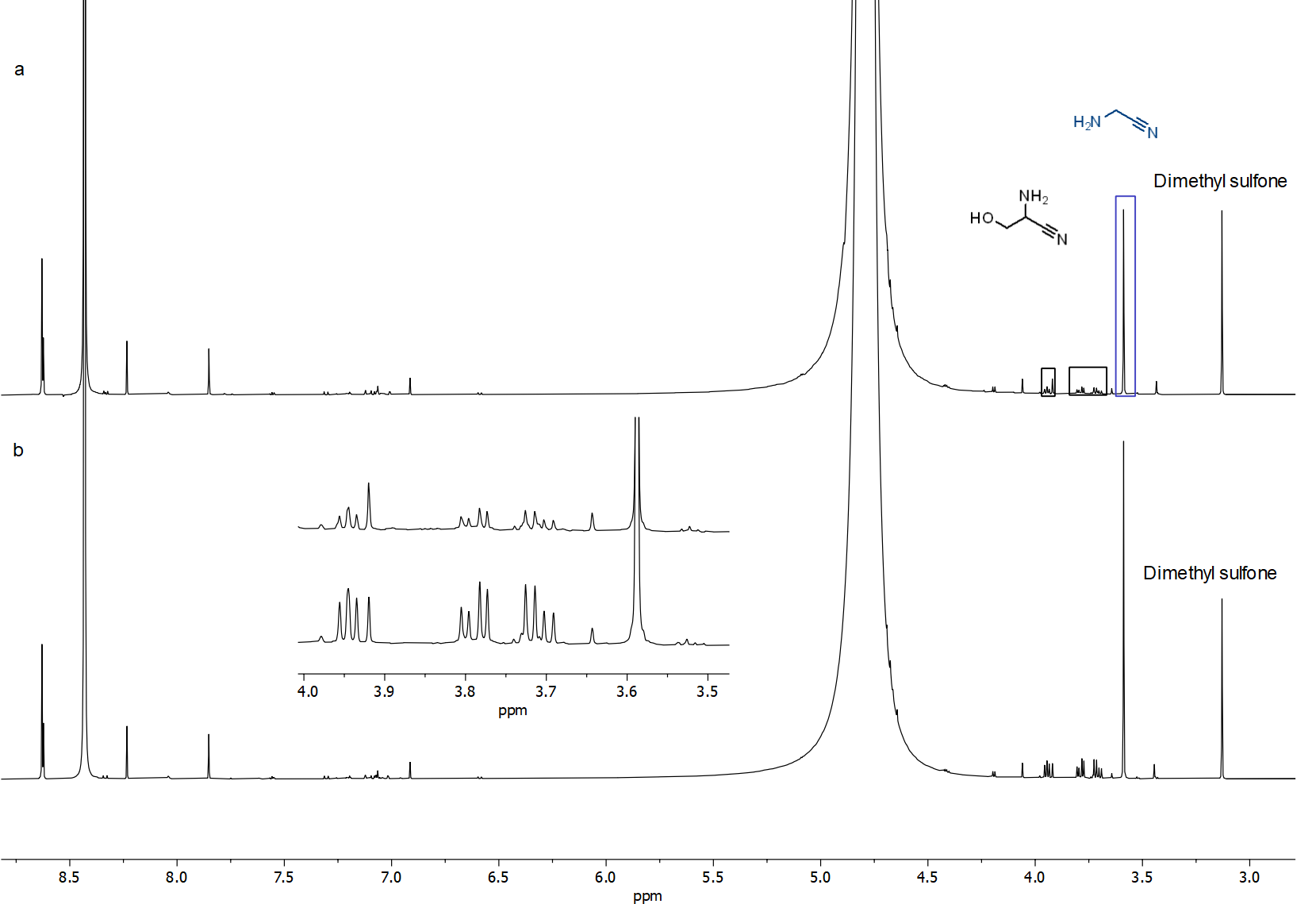


**Fig. S8. ^1^H-NMR spectra (500 MHz, D_2_O, 3.2–8.8 ppm) of prebiotically produced aminoacetonitrile 11 and 2-amino-3-hydroxypropanenitrile 12 from glycolaldehyde 3.** (a) Reaction mixture after treatment with hydroxylamine (**7**, 0.6 eq.) and formic acid (**5**) for 3 days at 50°C and pH 4 and basification with ammonia (**8**) to pH 9 (b) reaction mixture spiked with commercially available products **11** and **12**.

**Purine Precursors**

**Sodium 2-cyano-1-hydroxyethane-1-sulfonate (17@B)**

Synthetic reference:

In a 15 mL pressure tube, 3,3-diethoxypropanenitrile (1.50 mL, 10.0 mmol, 1.0 eq) was dissolved in TFA/H_2_O (3:1, 4 mL). After the addition of NaHSO_3_ (1.04 g, 10.0 mmol, 1.0 eq) the tube was quickly sealed to prevent SO_2_ escape. The reaction mixture was stirred at 70 °C for 18 h during which the product precipitated. After filtration, the residue was washed with EtOH (2 x 10 mL) and dried under high vacuum to afford sodium 2-cyano-1-hydroxyethane-1-sulfonate (**17@B**; 1.282 mg, 7.405 mmol, 65–74 %) as a white solid.

**^1^H-NMR** (500 MHz, DMSO-*d_6_*) *δ* = 6.31 (d, J = 6.0 Hz, 1H; OH), 4.13 (ddd, J = 9.6, 6.0, 3.2 Hz, 1H; SO_3_CH), 2.88 (dd, J = 17.0, 3.2 Hz, 1H; CH2), 2.60 (dd, J = 17.0, 9.6 Hz, 1H; CH2). **^13^C-NMR** (126 MHz, DMSO-*d_6_*) *δ* = 119.00 (CN), 78.5 (CH), 21.9 (CH2). **IR** (cm 1): 𝜈̃ = 3287 (w), 1414 (w), 1321 (w), 1280 (m), 1240 (s), 1183 (s), 1112 (s), 1051 (s), 1018 (m), 942 (w), 879 (w), 818 (w). **HRMS** (ESI+): calc.: [C_3_H_4_NNa_2_O_4_S]^+^ 195.9651, found: 195.9654. **Crystallographic data**: Fig. S17, Table S2.

**S12**. Prebiotic formation from 2-amino-3-hydroxypropanenitrile (**12)**:

2-Amino-3-hydroxypropanenitrile (**12**; 4 µmol, 1.0 eq.) was mixed with sodium nitrite (**1·Na**) (8 µmol, 2 eq.) in water at a concentration of 20 mm, with the pH adjusted to 2 using HCl. The mixture was shaken at 25 °C and 750 rpm for 1 hour in an Eppendorf ThermoMixer®.

Following this, NaHSO_3_ (12 µmol, 3 eq.) was added, and the mixture was shaken under the same conditions for an additional hour, resulting in a final concentration of 18.9 mm. A sample (50 µL) of the reaction mixture was diluted with D_2_O (550 µL) and analyzed by NMR spectroscopy using maleic acid as an internal standard and spiked with the synthetically obtained sodium 2-cyano-1-hydroxyethane-1-sulfonate (**17@B**) (Fig. S9). The yield of the product was found to be 15–22%.

**^1^H-NMR** (500 MHz, D_2_O) *δ* = 4.64 (dd, *J* = 7.8, 4.5 Hz, 1H, CH), 3.15 (dd, *J* = 17.3, 4.5 Hz, 1H, CH_2_a), 2.99 (dd, *J* = 17.3, 7.8 Hz, 1H, CH_2_b) ppm.


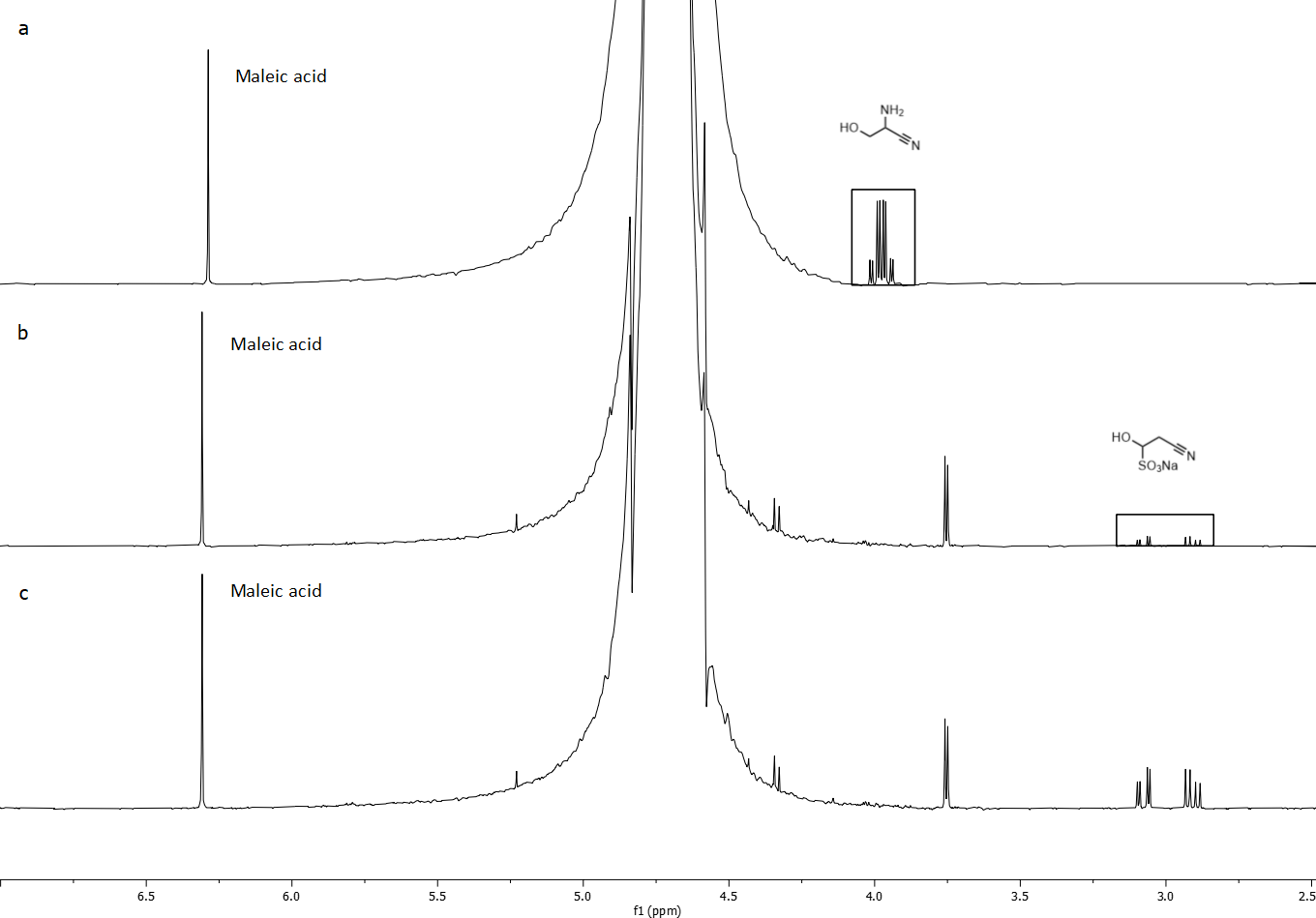


**Fig. S9. ^1^H-NMR spectra (500 MHz, D_2_O, 2.5–7.0 ppm) of prebiotically produced 2-cyano-1-hydroxyethane-1-sulfonate 17@B from 12.** (a) Starting material with maleic acid as an internal standard; (b) reaction mixture after sequential treatment with NaNO_2_ (**1·Na)** and NaHSO_3_ at pH 2 for 1 hour each; (c) reaction mixture spiked with synthetically obtained **17@B**.

**(1E,3E)-2-(hydroxyimino)malonaldehyde dioxime**

1,1,3,3-Tetramethoxypropane (10 mL, 60.71 mmol, 1.0 eq.) was mixed with HCl (33 mL, 66.8 mmol, 1.1 eq.) in a 250 mL round-bottom flask, resulting in a two-phase system. The mixture was stirred vigorously at room temperature for 30 minutes, forming a yellow solution. A solution of NaOAc (66.8 mmol, 1.1 eq.) in water (25 mL) was added dropwise with stirring until the pH reached 3.5. The reaction mixture was then placed in an ice bath, and a solution of NaNO_2_ (66.8 mmol, 1.1 eq.) in water (12.2 mL) was added dropwise while stirring continuously. The mixture was stirred at room temperature for an additional 30 minutes, during which the solution turned orange. Subsequently, a solution of NH_2_OH (151.9 mmol, 2.5 eq.) in water (9.3 mL) was added dropwise, and the mixture was stirred for another 50 minutes, leading to the formation of a suspension. The solid was isolated by filtration and washed with water (2 × 10 mL). The solid was then dried under vacuum, yielding 5.07 g (38.69 mmol, 64%) of (1E,3E)-2-(hydroxyimino)malonaldehyde dioxime.

**^1^H-NMR** (500 MHz, DMSO-*d_6_*) *δ* = 12.17 (br, 1H, HCONH), 11.90 (br, 1H, HCONH), 11.52 (br, 1H, HCONH), 8.14 (s, 1H, HONCH), 7.78 (s, 1H, HONCH) ppm. **^13^C-NMR** (126 MHz, DMSO-*d_6_*) *δ* = 145.99 (CNOH), 144.04 (CHNOH), 138.99 (CHNOH) ppm.

**Guanidinium salt of N-hydroxy-2-(hydroxyimino)acetimidoyl cyanide (19·G)**

Synthetic reference**:**

(1E,3E)-2-(hydroxyimino)malonaldehyde dioxime (7.631 mmol, 1 g, 1.0 eq.) and guanidine carbonate ((CH_5_N_3_)_2_·H_2_CO_3_; 3.815 mmol, 0.687 g, 0.5 eq.) were dissolved in water (38 mL) in a 250 mL round-bottom flask. The mixture was stirred overnight under a nitrogen atmosphere at 60°C. The next day, the solution appeared light orange. The mixture was then dried under vacuum at 55°C. The resulting slurry was stirred with acetone (30 mL) for 15 minutes and subsequently filtered. The solid was washed with acetone (3 × 30 mL) and dried under high vacuum to afford the guanidinium salt of N-hydroxy-2-(hydroxyimino)acetimidoyl cyanide (24; 0.533 g, 3.09 mmol, 40.5 %).

**^1^H-NMR** (500 MHz, DMSO-*d*_6_) *δ* = 8.15 (s, 1H, CH), 7.12 (s, 6H, guanidinium) ppm. **^13^C‑NMR** (126 MHz, DMSO-*d*_6_) *δ*= 157.94 (guanidinium), 148.53 (CH), 126.15 (C2), 114.14 (CN) ppm. **IR** (cm^‑1^): 𝜈̃ = 3417 (m), 3350 (m), 3127 (m), 2210 (m), 1661 (s), 1575 (m), 1429 (s), 1256 (m), 1132 (s), 970 (s), 924 (s), 802 (s), 676 (s). **HRMS** (ESI-): calc.: [C_3_H_2_N_3_O_2_]- 112.0152, found: 112.0140. **Elemental analysis:** Calc. (found) for C_4_H_10_N_6_O_3_: C, 27.91 (25.26); H, 4.68 (5.30); N, 48.82 (44.19). **Crystallographic data**: S20 and Table S1.

**N-Hydroxy-2-(hydroxyimino)acetimidoyl cyanide (19)**

**S13**. Prebiotic formation from sodium 2-cyano-1-hydroxyethane-1-sulfonate **17@B**:

Sodium 2-cyano-1-hydroxyethane-1-sulfonate (**17@B**; 20.0 µmol, 1.0 eq.) was mixed with NaNO_2_ (**1·Na**, 60 µmol, 3 eq.) in water at a concentration of 50 mm, with the pH adjusted to 2 using HCl. The mixture was shaken at 25 °C and 750 rpm for 2 hours, during which the pH increased to 5. A sample (50 µL) of the reaction mixture was diluted with D_2_O (550 µL) and analyzed by NMR spectroscopy using maleic acid as an internal standard (Fig. S10b).

Following this, hydroxylamine hydrochloride (87.5 µmol, 5 eq.) was added, and the mixture was shaken under the same conditions for an additional hour, resulting in a final concentration of 44.4 mm. During this time, the pH decreased to 2. A sample (50 µL) of the reaction mixture was diluted with D_2_O (550 µL) and analyzed by NMR spectroscopy (Fig. S10c) using maleic acid as an internal standard and spiked with the synthetically obtained guanidinium salt of N-hydroxy-2-(hydroxyimino)acetimidoyl cyanide (**19·G**) (Fig. S10d). The yield of the product was found to be 68–77%.


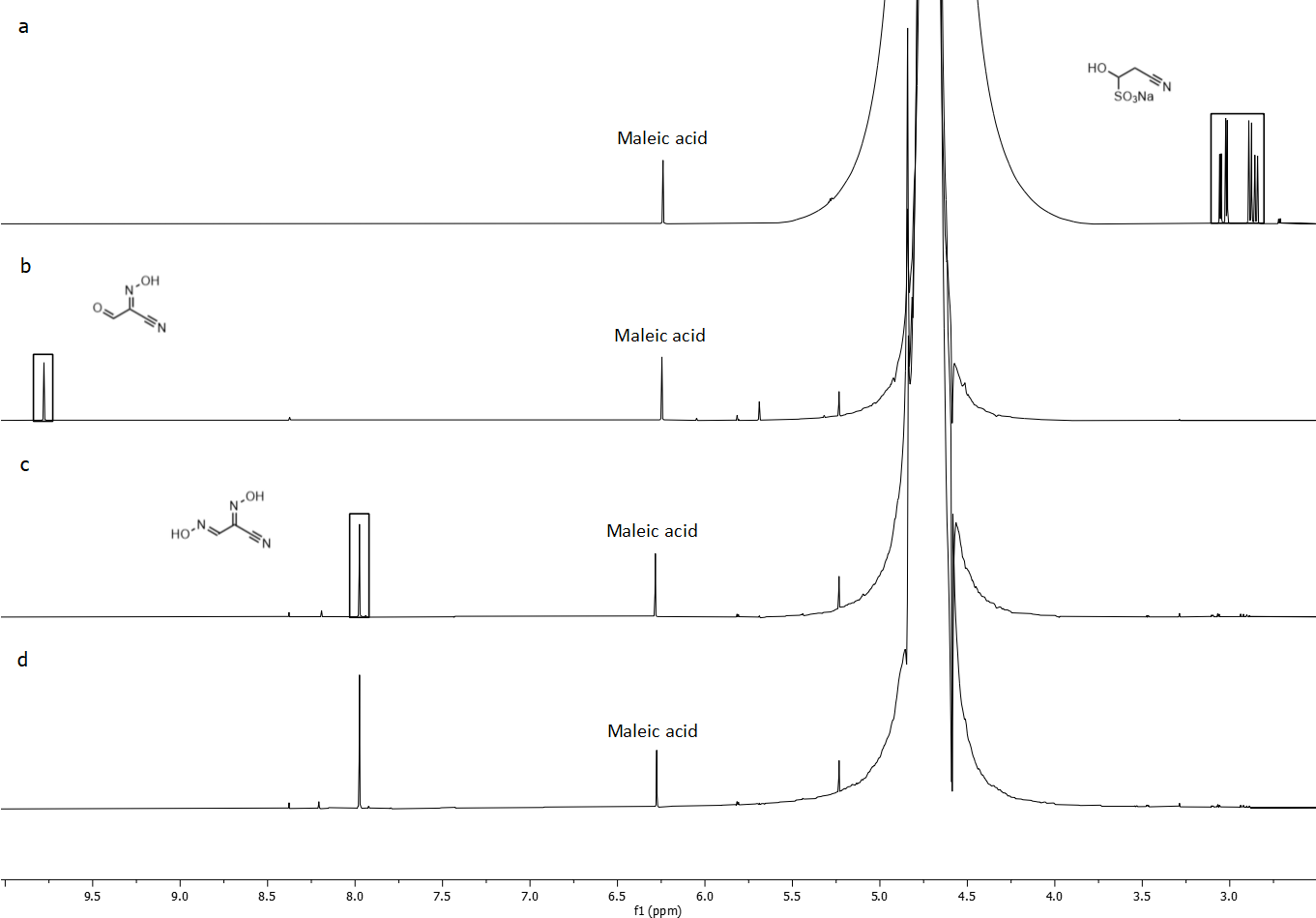


**Fig. S10. ^1^H-NMR spectra (500 MHz, D_2_O, 2–10 ppm) of prebiotically produced N-hydroxy-2-(hydroxyimino)acetimidoyl cyanide 19 from 17@B.** (a) Starting material with maleic acid as an internal standard; (b) reaction mixture after treatment with NaNO_2_ (**1·Na)** at pH 2–5 for 2 h with likely formation of N-hydroxy-2-oxoacetimidoyl cyanide **18**; (c) reaction mixture after NH_2_OH treatment at pH 2–5 for 1 hour; (d) reaction mixture spiked with synthetically obtained **19·G**.

**Hydroxycarbonimidoyl dicyanide (20·G)**

Synthetic reference:

Malononitrile (25.3 g, 38 mmol, 1.0 eq.) was dissolved in glacial acetic acid (35 mL) at room temperature. The solution was cooled to 0°C using an ice bath. To this cold solution, an aqueous solution of NaNO_2_ (46 g, 67 mmol, 1.76 eq.) in water (80 mL), pre-cooled to -5°C, was added dropwise with vigorous stirring. The temperature was maintained at 0°C for 2 hours using the ice bath, after which the mixture was stirred at room temperature for an additional 2 hours.

The reaction mixture was diluted with water and extracted with ethyl acetate (5 × 100 mL). The combined organic phases were washed with water, dried over sodium sulfate (Na_2_SO_4_), and concentrated under reduced pressure at 50°C to yield hydroxycarbonimidoyl dicyanide (**25**; 2.55 g, 26.8 mmol, 70%).

**^1^H-NMR** (500 MHz, DMSO-*d_6_*) *δ* = 6.90 (br, 6H, NH_2_) ppm. **^13^C-NMR** (126 MHz, DMSO-*d_6_*) *δ* = 119.12 (CN), 112.90 (CN), 106.72 (CNOH) ppm. **HRMS** (ESI-): calc.: [C_3_N_3_O]: 94.0046, found: 94.0034.

**S14**. Prebiotic formation from **19·G**:

Guanidinium salt of N-hydroxy-2-(hydroxyimino)acetimidoyl cyanide (**19·G**) was dissolved in water at a concentration of 50 mm, with the pH adjusted to 5 using HCl. The mixture was shaken at 50 °C and 750 rpm for 2 days in amount of 13–17%. The reaction mixture was analyzed by HPLC-MS, where the target compound was confirmed by spiking with synthetically obtained hydroxycarbonimidoyl dicyanide. The amount of the target compound was quantified using a calibration curve (Fig. S11).

**Fig. S11. HPLC-MS chromatograms (5–12 min, m/z: 94.0025–94.0039, 112.0129–112.0145) of prebiotically obtained hydroxycarbonimidoyl dicyanide (20·G).** (a) Chromatogram of the starting material (**19·G**); (b) reaction mixture after 2 days of shaking at 50°C and pH 5; (c) reaction mixture spiked with synthetically obtained hydroxycarbonimidoyl dicyanide **20·G**.

**Pyrimidine Precursors**

**Isoxazol-5-amine (21)**

**S15**. Prebiotic formation from sodium 2-cyano-1-hydroxyethane-1-sulfonate (**17@B**):

Sodium 2-cyano-1-hydroxyethane-1-sulfonate (**17@B**; 20.0 µmol, 1.0 eq.) was mixed with hydroxylamine hydrochloride (160 µmol, 8 eq.) in water at a concentration of 50 mm, with the pH adjusted to 6–7 using NaOH. The mixture was shaken at 25 °C and 750 rpm overnight in an Eppendorf ThermoMixer®. A sample (100 µL) of the reaction mixture was diluted with D_2_O (500 µL) and analyzed by NMR spectroscopy using maleic acid as an internal standard and spiked with commercial isoxazol-5-amine (**21**) to confirm product formation (Fig. S12). The yield of the product was found to be quantitative.

**^1^H-NMR** (500 MHz, D_2_O) *δ* = 8.13 (d, *J* = 2.2 Hz, 1H, CHNO), 5.25 (d, *J* = 2.2 Hz, 1H, CH) ppm.


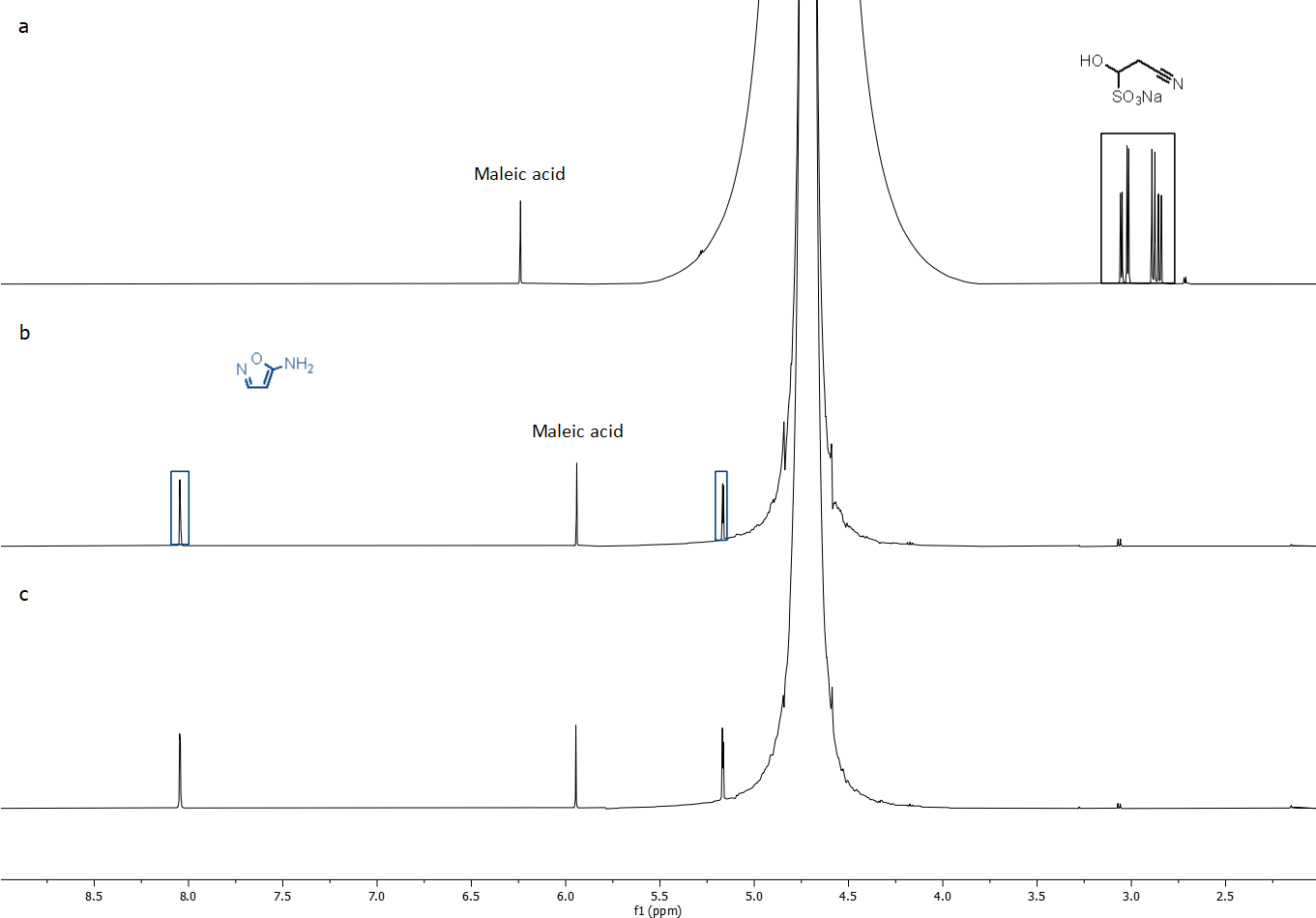


**Fig. S12. ^1^H-NMR spectra (500 MHz, D_2_O, 2.0–9.0 ppm) of prebiotically produced isoxazol-5-amine 21 from 17@B.** (a) Starting material with maleic acid as an internal standard; (b) reaction mixture after treatment with hydroxylamine at pH 6–7 at 25 °C overnight; (c) reaction mixture spiked with commercially available isoxazol-5-amine **21**.

**S16.** Prebiotic one-pot formation of **19** and **21** from 2-cyano-1-hydroxyethane-1-sulfonate (**17@B**):

Sodium 2-cyano-1-hydroxyethane-1-sulfonate (**17@B**, 50 µmol, 8.6 mg, 1.0 eq.) was dissolved in H_2_O (50 µL). Formic acid (**5**, 1 wt% in H_2_O, 100 µL, pH 4) and sodium nitrite (**1·Na**) (62.5 µL, 62.5 µmol, 1.25 eq.) were added resulting in a 240 mm concentration of **17@B**. The reaction mixture was shaken at 25 °C and 750 rpm for 1h in an Eppendorf ThermoMixer®. Hydroxylamine (**7**, 62.5 µL, 62.5 µmol 1.25 eq.) was added and the mixture was shaken for a further 1h. A sample (50 µL) of the reaction mixture in D_2_O (500 µL) was analyzed by NMR spectroscopy using maleic acid as an internal standard and spiked the product with **19·G** and **21** (Fig. S13). The concurrent one-pot formation of isoxazole-5-amine (**21**) in 17-18% yield and N-hydroxy-2-(hydroxyimino)acetimidoyl cyanide (**19**) in 24-27% yield was observed.

*
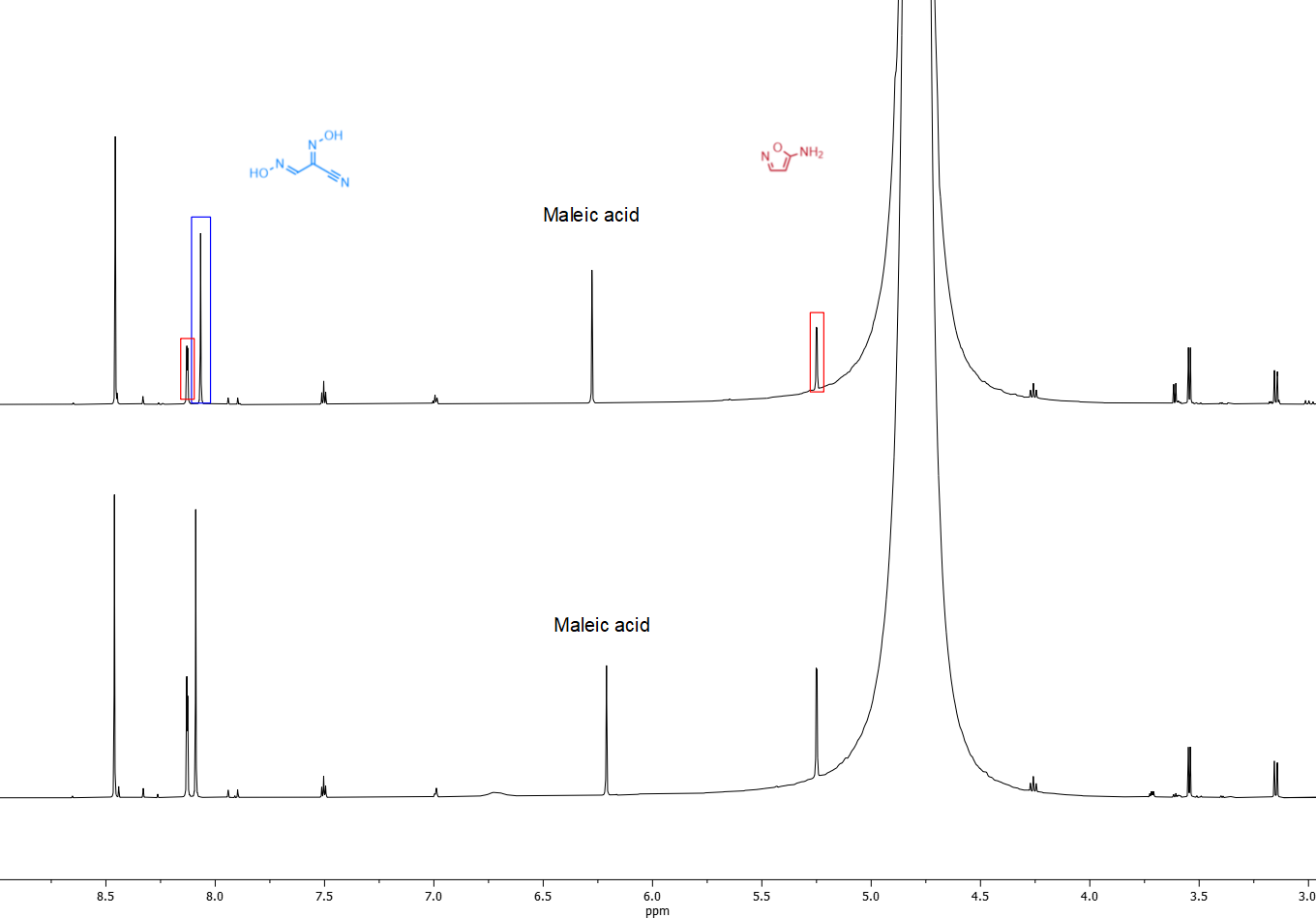
*

**Fig. S13. ^1^H-NMR spectra (500 MHz, D_2_O, 3.0–9.0 ppm) of one-pot prebiotically produced N-hydroxy-2-(hydroxyamino)acetimidoyl cyanide (19) and isoxazol-5-amine (21) from 2-cyano-1-hydroxyethane-1-sulfonate 17@B.** (a) Reaction mixture after treatment with nitrite (**1**) and formic acid (**5**) for 1h and subsequent treatment with hydroxylamine (**7**) for another 1h for at 25°C and pH 4 (b) reaction mixture spiked with **19·G** and **21**.

**(1Z,3E)-N'-hydroxy-3-(hydroxyimino)propanimidamide (22)**

**Synthetic reference:**

Sodium 2-cyano-1-hydroxyethane-1-sulfonate (50 mg, 0.243 mmol, 1 eq.) was suspended in 100 µL of H₂O. A mixture of hydroxylamine (2.425 mmol, 10 eq) and sodium hydroxide (30 0.3 mmol, 1.24 eq.) was added to the suspension. The mixture was stirred at room temperature for 30 minutes, maintaining the pH at 9.

After this, reaction mixture was extracted with EtOAc (8 x 10 mL). The combined organic layers were dried over Na_2_SO_4_, filtered and the solvent removed *in vacuo.* The yield of (1Z,3E)-N'-hydroxy-3-(hydroxyimino)propanimidamide (**22**) was found to be 10% (20 mg, 0.024 mmol mmol) as a colorless oil.

NMR analysis revealed an isomeric ratio of 55 (*1Z, 3E*) : 45 (*1Z, 3Z*).

**^1^H-NMR** (500 MHz, DMSO-*d_6_*) *δ* = 10.99 (br, (*1Z, 3E*), 1H, CHNOH), 10.54 (*1Z, 3Z*), 1H, CHNOH), 8.95 (br, (*1Z, 3E*), 1H, CNH_2_NOH), 8.95 (br, (*1Z, 3Z*), 1H, CNH_2_NOH), 7.32 (t, (*1Z, 3E*), *J* = 6.2 Hz, 1H, CH), 6.78 (t, (*1Z, 3Z*), *J* = 4.9 Hz, 1H, CH), 5.45 (br, (*1Z, 3Z*), 2H, NH_2_), 5.49 (br, (*1Z, 3E*), 2H, NH_2_), 2.99 (d, *(1Z, 3E)*, *J* = 4.9 Hz, 2H, CH_2_), 2.84 (d, (*1Z, 3Z*), J = 6.2 Hz, 2H, CH_2_) ppm. **^13^C-NMR** (126 MHz, DMSO-*d_6_*) *δ* = 150.11 ((*1Z, 3Z*), CNH_2_), 149.87 ((*1Z, 3E*), CNH_2_), 146.36 ((*1Z, 3Z*), CH), 146.11 ((*1Z, 3E*), CH), 31.94 ((1Z, 3Z), CH_2_), 27.88 ((1Z, 3E), CH_2_) ppm.

**Sodium 1,3-diamino-3-(hydroxyimino)propane-1-sulfonate (23@B)**

Synthetic reference:

Sodium 2-cyano-1-hydroxyethane-1-sulfonate (50 mg, 0.243 mmol, 1 eq.) was suspended in 100 µL of H₂O. A mixture of hydroxylamine (2.425 mmol, 10 eq) and sodium hydroxide (30 0.3 mmol, 1.24 eq.) was added to the suspension. The mixture was stirred at room temperature for 30 minutes, maintaining the pH at 9.

After this, NaHSO₃ (1.2 mmol, 5 eq.) and 200 µL of 37% HCl were added simultaneously. The resulting mixture was stirred for an additional 30 minutes, during which the pH was adjusted to 1.

The reaction mixture was then added dropwise to 10 mL of acetone under stirring. The precipitate formed was filtered off and dried overnight under high vacuum to afford sodium 1,3-diamino-3-(hydroxyimino)propane-1-sulfonate (**23@B**; 99.4 mg, 0.07 mmol, 28 %) as a white solid.

**^1^H-NMR** (500 MHz, DMSO-*d_6_*) *δ* = 4.73 (dd, *J* = 15.0, 5.5 Hz, 1H, SO_3_CH), 3.30 (dd, *J* = 15.0, 8.7 Hz, 1H, CH_2_a), 3.02 (dd, *J* = 8.7, 5.5 Hz, CH_2_b) ppm.

**^13^C-NMR** (126 MHz, DMSO-*d_6_*) *δ* = 160.67 (CNH_2_NOH), 66.96 (CH), 31.89 (CH_2_) ppm.

**3,3-Diethoxy-N'-hydroxypropanimidamide**

The 3,3-diethoxypropanenitrile (1.50 mL, 10.0 mmol, 1.0 eq) was dissolved in MeOH/H_2_O (1:1, 10 mL). After the addition of Na_2_CO_3_ (2.65 g, 25 mmol, 2.5 eq) and NH_2_OH·HCl (2,431 g, 35 mmol, 3.5 eq.) the mixture was stirred at 55 °C for 18 h. After that, water (10 mL) was added and mixture was extracted with EtOAc (8 x 10 mL). The combined organic layers dried over Na_2_SO_4_, filtered and the solvent removed *in vacuo.* The yield of 3,3-diethoxy-N'-hydroxypropanimidamide was found to be 93–100% (1.282 mg, 7.405 mmol) as a colorless oil.

**^1^H-NMR** (500 MHz, DMSO-*d_6_*) *δ* = 8.91 (br, 1H, NOH), 5.29 (br, 1H, NH_2_), 4.74 (t, *J* = 5.7 Hz, 1H, CH), 3.59 (dq, J = 9.5, 7.0 Hz, 2H, CH_3_CH_2_a, 3.43 (dq, J = 9.5, 7.0 Hz, 2H, CH_3_CH_2_b), 2.21 (d, J = 5.7 Hz, 2H, CH_2_), 1.10 (t, J= 7.0 Hz, 6H, CH_3_) ppm. **^13^C-NMR** (126 MHz, DMSO-*d_6_*) *δ* =148.86 (CNOHNH_2_), 100.53 (CH), 61.13(CH_3_CH_2_), 36.44 (CH_2_), 15.69 (CH_3_) ppm.

**Sodium-3-amino-1-hydroxy-3-(hydroxyimino)propane-1-sulfonate (24@B)**

Synthetic reference:

In a 15 mL pressure tube, 3,3-diethoxy-N’-hydroxypropanimidamide (1.50 mL, 10.0 mmol, 1.0 eq) was dissolved in TFA/H_2_O (3:1, 4 mL). After the addition of NaHSO_3_ (1.04 g, 10.0 mmol, 1.0 eq) the tube was quickly sealed to prevent SO_2_ escape. The reaction mixture was stirred at r.t. for 48 h during which the product precipitated. After filtration, the residue was washed with EtOH (2 x 10 mL) filtered and the solvent removed *in vacuo.* The yield of sodium-3-amino-1-hydroxy-3-(hydroxyimino)propane-1-sulfonate was found to be 51–55% (**24@B**; 804 mg, 4.64 mmol) as a white solid.

**^1^H-NMR** (500 MHz, DMSO-*d_6_*) *δ* = 10.68 (br, 1H, NOH), 8.67 (br, 2H, NH_2_), 6.10 (br, 1H, OH), 4.32 (dd, *J* = 9.9. 3.6 Hz, 1H, SO_3_CH), 2.85 (dd, *J* = 14.4, 3.6 Hz, 1H, CH_2_a), 2.55 (dd, *J* = 14.4, 9.9 Hz, 1H, CH_2_b) ppm. **^13^C-NMR** (126 MHz, DMSO-*d_6_*) *δ* = 160.88 (CNOHNH_2_), 79.36 (SO_3_CH), 32.38 (CH_2_) ppm.

**Isoxazol-3-amine (25)**

**S17.** Prebiotic formation mixture of **23@B** and **24@B** from sodium 2-cyano-1-hydroxyethane-1-sulfonate (**17@B**):

Sodium 2-cyano-1-hydroxyethane-1-sulfonate (**17@B**; 40.0 µmol, 1.0 eq.) was mixed with hydroxylamine hydrochloride (160 µmol, 8 eq.) in water at a concentration of 55.6 mm, with the pH adjusted to 9 using NaOH. The mixture was shaken at 25 °C and 750 rpm overnight in an Eppendorf ThermoMixer®. A sample (300 µL) of the reaction mixture was taken, diluted with D_2_O (300 µL), and analyzed by NMR spectroscopy. The formation of the products was confirmed using synthesized or commercially purchased standards and quantified with maleic acid and DMSO_2_ as internal standards (Fig. S14b).

Subsequently, NaHSO_3_ (160 µmol, 4 eq.) was added, and the pH was adjusted to 5 using HCl, resulting in a concentration of 34.3 mm. The mixture was shaken at 25 °C and 750 rpm for 6 hours, during which the pH changed to 2–3. A sample (300 µL) of the reaction mixture was taken, diluted with D_2_O (300 µL), and analyzed by NMR spectroscopy. The formation of the products was confirmed again with synthesized or commercially purchased standards and quantified using maleic acid as the internal standard (Fig. S14c). The yields of sodium (Z)-1,3-diamino-3-(hydroxyimino)propane-1-sulfonate **23@B** and sodium (Z)-3-amino-1-hydroxy-3-(hydroxyimino)propane-1-sulfonate **24@B** were found to be 21-35% and 10-13% correspondently.


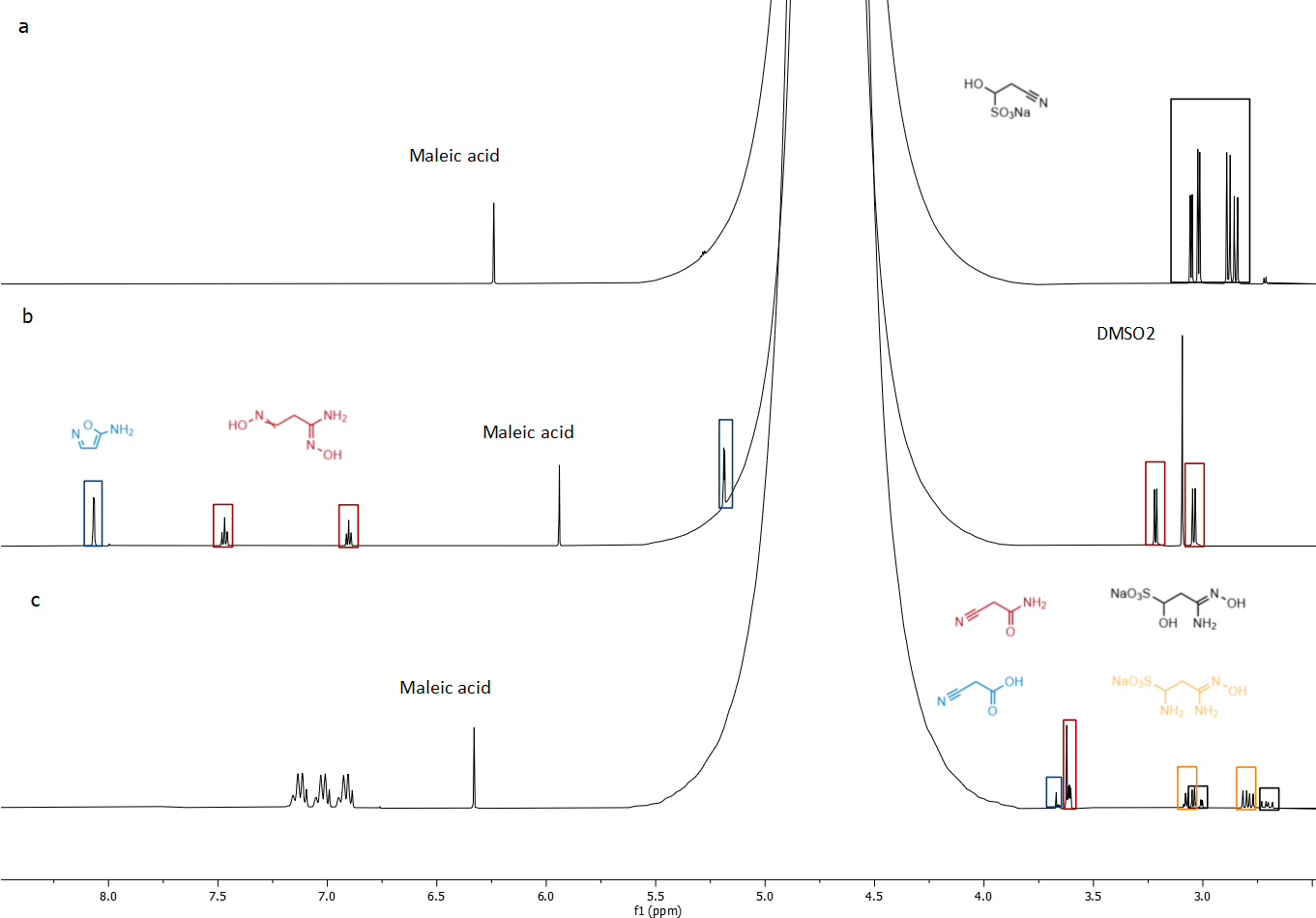


**Fig. S14. ^1^H-NMR spectra (500 MHz, D_2_O, 2.0–9.0 ppm) of prebiotically produced isoxazol-3-amine 25.** (a) Starting material **17@B** with maleic acid as an internal standard; (b) reaction mixture after treatment with hydroxylamine at pH 9 overnight; (c) reaction mixture after NaHSO_3_ treatment at pH 2–5 for 6 hours.

**S18**. Prebiotic formation from mixture of **23@B** and **24@B**:

The mixture of **23@B** (18,75 µmol, 3 eq.) and **24@B** (6,25 µmol, 1 eq.) in 500 µL of water was shaken at 70 °C and 750 rpm for 7 days at pH 2. The samples of the reaction mixture were taken in 3 days (150 µL, Fig. S15b) after reaction beginning and after 7 days (50 µL, Fig. S15c), diluted with D_2_O (550 µL), and analyzed by NMR spectroscopy using maleic acid as an internal standard, and spiked with commercially purchased compounds to confirm the observed substances. The yield of isoxazol-3-amine (**25**) after 7 days was found to be 35-40 %.

**^1^H-NMR** (500 MHz, D_2_O) *δ* = 8.28 (d, *J* = 1.9 Hz, 1H, CHON), 6.14 (d, *J* = 1.9 Hz, 1H, CH) ppm.


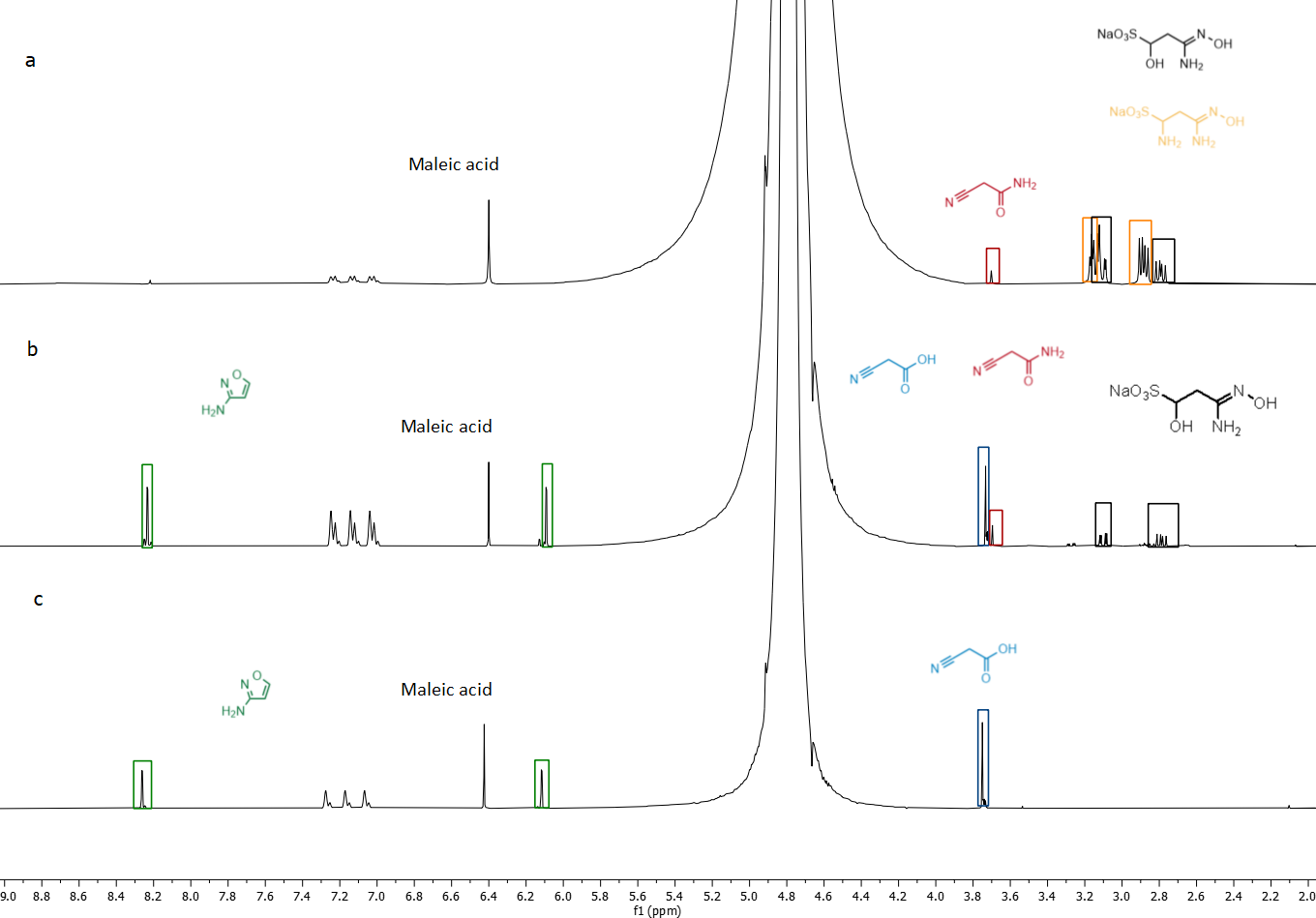


**Fig. S15. ^1^H-NMR spectra (500 MHz, D_2_O, 2.0–9.0 ppm) of prebiotically produced isoxazol-3-amine 25.** (a) Starting mixture of **23@B** and **24@B** (3:1) with maleic acid as an internal standard; (b) reaction mixture after heating at 70 °C for 3 days at pH 2; (c) reaction mixture after heating at 70 °C for 7 days at pH 2.

**Crystallographic Data**


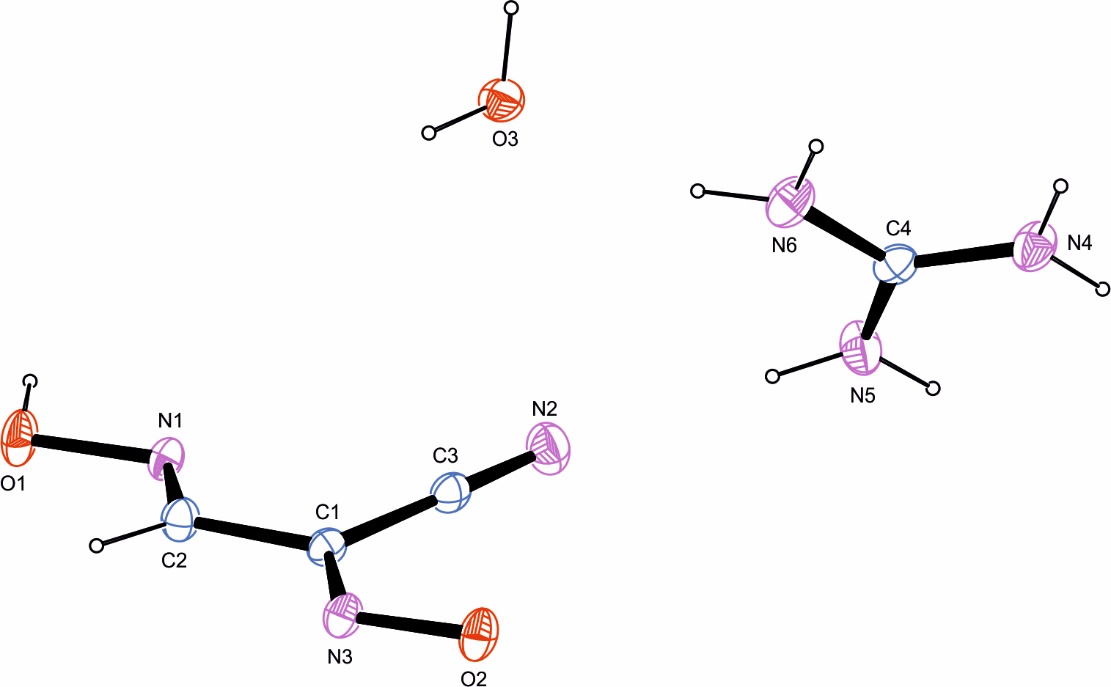


**Fig. S16. Crystal structure of the guanidinium salt of N-hydroxy-2-(hydroxyimino)acetimidoyl cyanide (19·G).** Nitrogen atoms (pink), oxygen atoms (red) and carbon atoms (blue) are represented by large spherical structures. Hydrogen atoms (white) are represented by small spherical structures.


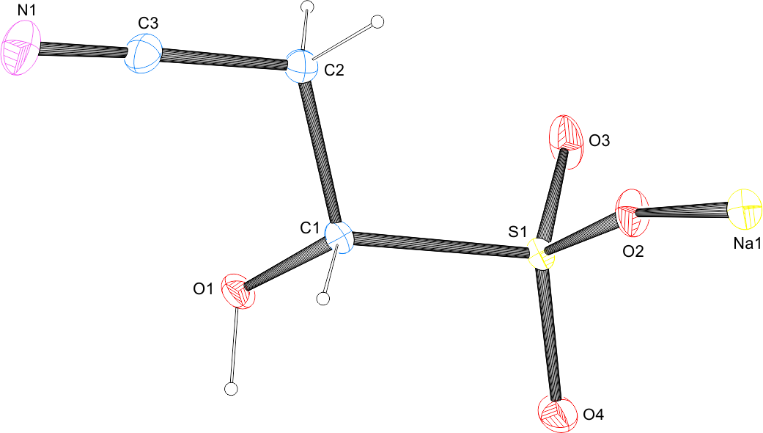


**Fig. S17. Crystal structure of sulfonate 17@B.** Nitrogen atoms (pink), oxygen atoms (red), sulfur/sodium atoms (yellow) and carbon atoms (blue) are represented by large spherical structures. Hydrogen atoms (white) are represented by small spherical structures.

**Table S1:** Crystallographic data for the guanidinium salt of N-hydroxy-2-(hydroxyimino)acetimidoyl cyanide (**19·G**)

| net formula | C_8_H_18_N_12_O_5_ |
| --- | --- |
| *M*_r_/g mol^−1^ | 362.34 |
| crystal size/mm | 0.120 × 0.080 × 0.070 |
| *T*/K | 173.(2) |
| radiation | MoKα |
| diffractometer | 'Bruker D8 Venture TXS' |
| crystal system | monoclinic |
| space group | 'P 1 2/n 1' |
| *a*/Å | 7.6411(7) |
| *b*/Å | 8.1977(8) |
| *c*/Å | 13.4853(14) |
| α/° | 90 |
| β/° | 96.633(4) |
| γ/° | 90 |
| *V*/Å^3^ | 839.06(14) |
| *Z* | 2 |
| calc. density/g cm^−3^ | 1.434 |
| μ/mm^−1^ | 0.119 |
| absorption correction | Multi-Scan |
| transmission factor range | 0.95–0.99 |
| refls. measured | 14355 |
| *R*_int_ | 0.0513 |
| mean σ(*I*)/*I* | 0.0323 |
| θ range | 3.658–27.473 |
| observed refls. | 1613 |
| *x, y* (weighting scheme) | 0.0273, 0.3864 |
| hydrogen refinement | mixed |
| Flack parameter | ? |
| refls in refinement | 1927 |
| parameters | 146 |
| restraints | 0 |
| *R*(*F*_obs_) | 0.0356 |
| *R*_w_(*F*^2^) | 0.0889 |
| *S* | 1.060 |
| shift/error_max_ | 0.001 |
| max electron density/e Å^−3^ | 0.185 |
| min electron density/e Å^−3^ | −0.160 |

**Table S2:** Crystallographic data for sulfonate **17@B**

| net formula | C_3_H_4_NNaO_4_S |
| --- | --- |
| *M*_r_/g mol^−1^ | 173.12 |
| crystal size/mm | 0.090 × 0.070 × 0.040 |
| *T*/K | 173.(2) |
| radiation | MoKα |
| diffractometer | 'Bruker D8 Venture TXS' |
| crystal system | monoclinic |
| space group | 'P 1 21/c 1' |
| *a*/Å | 9.2682(3) |
| *b*/Å | 9.2707(3) |
| *c*/Å | 7.3226(2) |
| α/° | 90 |
| β/° | 93.3640(10) |
| γ/° | 90 |
| *V*/Å^3^ | 628.09(3) |
| *Z* | 4 |
| calc. density/g cm^−3^ | 1.831 |
| μ/mm^−1^ | 0.532 |
| absorption correction | Multi-Scan |
| transmission factor range | 0.95–0.98 |
| refls. measured | 12172 |
| *R*_int_ | 0.0308 |
| mean σ(*I*)/*I* | 0.0174 |
| θ range | 3.111–28.281 |
| observed refls. | 1426 |
| *x, y* (weighting scheme) | 0.0281, 0.4425 |
| hydrogen refinement | mixed |
| Flack parameter | ? |
| refls in refinement | 1547 |
| parameters | 95 |
| restraints | 0 |
| *R*(*F*_obs_) | 0.0257 |
| *R*_w_(*F*^2^) | 0.0696 |
| *S* | 1.115 |
| shift/error_max_ | 0.001 |
| max electron density/e Å^−3^ | 0.541 |
| min electron density/e Å^−3^ | −0.371 |
